# Zinc-based Nanomaterials in Cancer Therapy: Mechanisms, Applications, and Future Directions

**DOI:** 10.7150/thno.117773

**Published:** 2025-07-11

**Authors:** Sen Mu, Hongyu Yang, Siqi Wang, Aiyang Tong, Rui Ding, Jiaxin Wang, Dongkai Wang, Ji Li

**Affiliations:** Department of Pharmaceutics, School of Pharmacy, Shenyang Pharmaceutical University, No. 103, Wenhua Road, Shenyang, 110016, P. R. China.

**Keywords:** Zn-based nanomaterials, Oxidative stress, Cancer therapy, Multimodal treatment, Tumor microenvironment

## Abstract

Metabolic reprogramming of cancer cells has resulted in a preference for aerobic glycolysis over oxidative phosphorylation, leading to increased energy demand and elevated oxidative stress. Zinc ions and Zn-based nanomaterials show promise in targeting these metabolic changes and enhancing cancer therapies. Zn-based nanomaterials (e.g., ZnO₂, ZnO, ZIF-8, ZnS) disrupt tumor energy metabolism and induce oxidative stress via Zn²⁺ release in the tumor microenvironment, thereby promoting metabolic dysfunction, apoptosis, and focal cell death. These materials can also be integrated into multimodal therapies for synergistic effects, including photodynamic, acoustic, gaseous, and immunotherapy. This review discusses the mechanisms of Zn-based nanomaterials disrupting tumor metabolism and inducing oxidative stress, their applications in cancer therapy, and future directions for development. By systematically summarizing the research progress of Zn-based nanomaterials, we aim to provide new ideas and strategies for cancer therapy.

## Introduction

During the reprogramming of cellular metabolism, cancer cells preferentially utilize aerobic glycolysis over mitochondrial respiration to meet their heightened nutritional and energy demands, which are associated with rapid proliferation 1. In response to changes in the microenvironment, cancer cells' metabolic flexibility and adaptability allow them to dynamically transition between aerobic glycolysis and mitochondrial respiration, guaranteeing an adequate supply of adenosine triphosphate (ATP) and vital nutrients 2. Furthermore, the highly active energy metabolism characteristic of tumors frequently leads to the accumulation of reactive oxygen species (ROS). Elevated hydrogen peroxide (H₂O₂) levels can function as secondary messengers, rapidly modulating cellular metabolic pathways by oxidizing cysteine residues in various metabolic enzymes 3. The remodeled tumor metabolism subsequently upregulates antioxidants, including glutathione (GSH), to maintain redox homeostasis and prevent cell death caused by excessive oxidative stress. This tight interdependence between metabolic and oxidative signals establishes a critical metabolic-redox loop that not only confers survival advantages to tumors but also strategically presents unique therapeutic targets for metal ion-mediated interventions 4.

Ions (e.g., Zn²⁺, Ga²⁺, K⁺, Na⁺, Cu²⁺, etc.) are crucial for numerous biological functions, such as modulation of intracellular communication, maintaining stable intracellular osmotic pressure, immune cell activation, and mediating inflammatory responses 5. Recently, significant research has been done into the effectiveness of metal-based biomaterials as immunomodulators. These materials show promise in boosting anti-tumor immune responses and reversing immune suppression, all while minimizing adverse effects 6. For instance, Mn²-mediated sensitization to cytoplasmic DNA and STING activation can be integrated with gas therapy, photothermal therapy (PTT), sonodynamic therapy (SDT), and drug administration 7. Ca²⁺ can mediate mitochondrial damage and ROS generation, which in turn activate caspase-3 and cleave GSDME, causing tumor cell death 8. While these metal ions exhibit specific therapeutic effects, Zn²⁺ stands out due to its multifaceted and self-amplifying roles in disrupting cancer metabolism and redox homeostasis, a feature unmatched by other biologically relevant metals. In the human body, Zn²⁺ exists as a divalent cation and is the second most abundant essential trace element as well as a cofactor for many enzymes 9. Research has shown that increased levels of Zn²⁺ can suppress the function of critical metabolic enzymes, including glyceraldehyde-3-phosphate dehydrogenase, phosphofructokinase, lactate dehydrogenase (LDH), and α-ketoglutarate dehydrogenase (α-KGDHC), while also interfering with the mitochondrial electron transport chain (ETC) 10-12. Additionally, Zn²⁺ has been found to promote the generation of ROS. This occurs through mechanisms such as the single-electron reduction of O₂ in the ETC, producing superoxide anions (O₂⁻), or the single- or double-electron reduction of O₂ facilitated by α-KGDHC and nicotinamide adenine dinucleotide phosphate oxidase, resulting in the formation of H₂O₂ 13. Consequently, increasing Zn²⁺ levels offers a promising approach to targeting the energy-metabolism-redox loop. Furthermore, elevated Zn²⁺ levels induce the upregulation of cysteine-rich metallothionein (MT), which plays a crucial role in regulating intracellular Zn²⁺ balance and alleviating oxidative stress. Consequently, the upregulation of MT levels attenuates the inhibitory effects of Zn²⁺ on metabolism and alleviates oxidative stress-induced damage 14. Notably, several studies have demonstrated that elevated ROS levels can oxidize MT, disrupt Zn²-cysteine binding, trigger Zn²⁺ release, and compromise Zn²⁺ homeostasis 15, 16. Therefore, upsetting the equilibrium between Zn²⁺ and ROS in cells can initiate an uncontrollable self-amplifying loop and significantly impair the ability of tumor cells to respond to external stimuli.

Leveraging these biological properties, zinc-based nanomaterials (ZIF-8, ZnO, ZnO₂, ZnS) exhibit TME-responsive characteristics with broad applications in cancer therapy, including chemotherapy, PDT, SDT, piezoelectric therapy, immunotherapy, and gas therapy 17. These nanomaterials can efficiently penetrate tumor cells, release Zn²⁺, and disrupt metabolic processes and DNA replication in tumor cells, ultimately inducing cell cycle arrest and apoptosis. Additionally, the photothermal effect of some zinc ion nanomaterials (e.g., ZnO, ZIF-8) can generate localized heat under laser irradiation, selectively ablating tumor cells while preserving adjacent healthy tissues Their photocatalytic activity can generate ROS, which disrupt the antioxidant defense systems of tumor cells, thereby potentiating the efficacy of chemotherapeutic agents 18. Therefore, Zn-based nanomaterials hold considerable promise for enhancing the efficacy of existing cancer therapies while minimizing adverse systemic effects and improving their therapeutic precision in practice.

Numerous Zn-based nanomaterials have been developed and used widely in cancer therapy due to the special benefits of Zn²⁺ 19-21. The purpose of this review is to present an in-depth investigation of the latest developments in cancer treatment as well as the uses of Zn-based nanomaterials. First, it outlines Zn²⁺ signaling mechanisms and associated proteins in both intracellular and tumor microenvironmental contexts. The specific mechanisms underlying Zn-induced tumor cell apoptosis are elucidated through a thorough analysis of these signaling pathways and protein functions. Subsequently, it reviews Zn-based nanomaterials with TME-responsive properties, including ZnO₂, ZnO, ZIF-8, and ZnS, and their specific applications in various cancer treatment modalities, such as chemotherapy, PDT, SDT, piezoelectric therapy, immunotherapy, and gas therapy, while analyzing the advantages and mechanisms of each material. Additionally, it summarizes recent advancements in other Zn-based materials, encompassing innovations in synthesis methods, performance optimization, and novel application exploration. Finally, it provides an in-depth discussion of the current challenges associated with Zn-responsive nanomaterials and proposes prospects and development directions, aiming to offer valuable insights and guidance for further research.

## Multifaceted Role of Zinc Ions in Cell Physiology and Pathology

### Critical role of zinc ions in cell physiology and signaling

**Figure 1:**
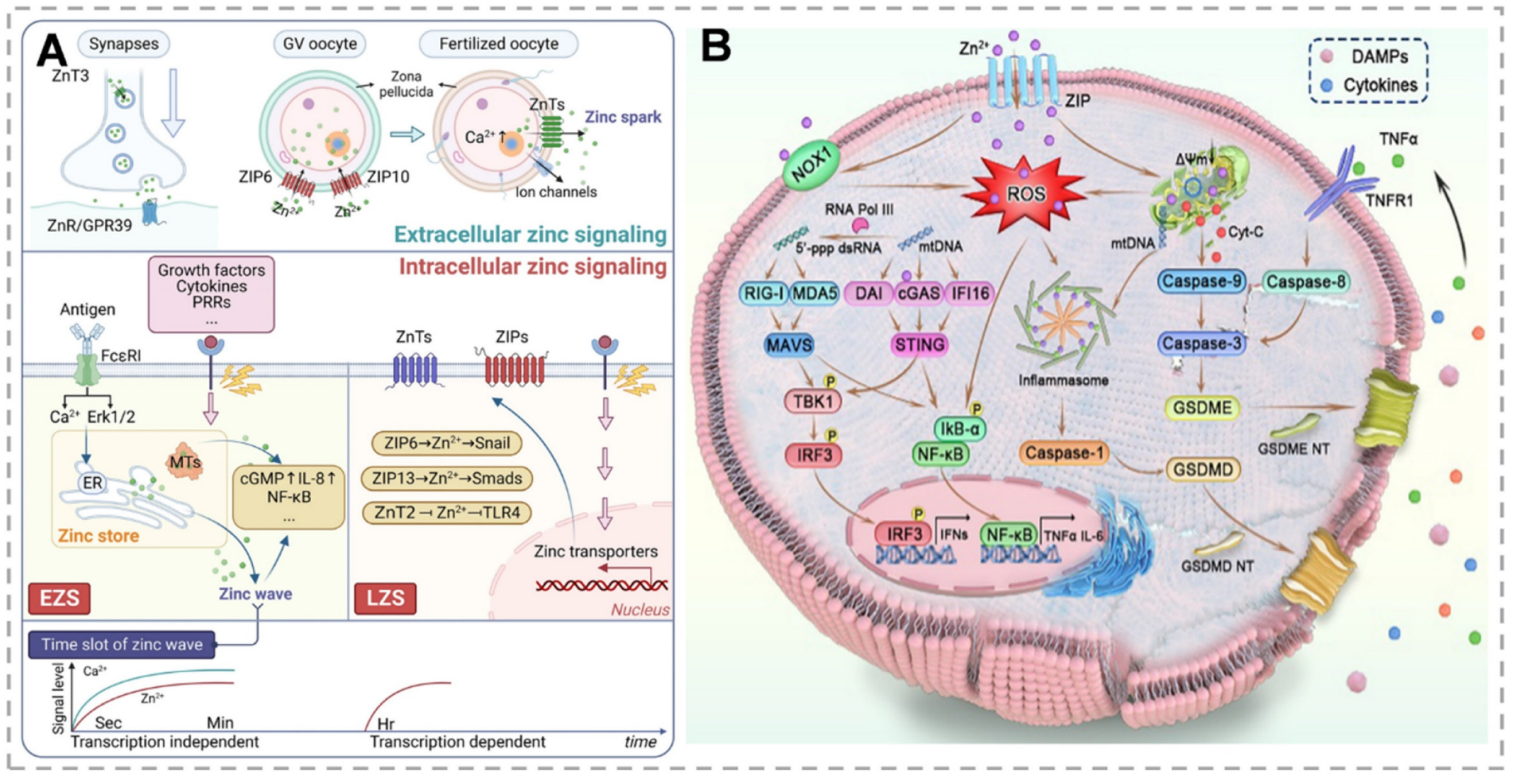
(A) Complex Role of Zinc Signaling in Cellular Processes and Associated Pathways. Zn²⁺ as an intracellular second messenger mediates inflammatory signaling and immune homeostasis by activating downstream pathways via EZS and LZS, whose conductance is dependent on the ZnT, ZIP family, and MT modulation, and which play key roles in cellular communication and signaling. Zn²⁺ as an intracellular second messenger activates downstream pathways via EZS and LZS and plays a critical role in cellular communication and signaling. (B) Mechanism of zinc ion-induced apoptosis and pyroptosis in tumor cells. Activation of the caspase pathway through mitochondrial damage triggers apoptosis, while activation of inflammatory vesicles cleaves Gasdermin proteins to induce pyroptosis, both of which synergistically release inflammatory factors to enhance anti-tumor immunity. **(A)** Reproduced with permission from 29, copyright 2025, Springer Nature Limited. **(B)** Reproduced with permission from 47, copyright 2024, Chinese Chemical Society.

Zn²⁺, an essential biological metal ion, is vital for cellular biochemistry and nutrition, facilitating cell growth and proliferation. Within cells, Zn²⁺ exists mainly in two forms: protein-bound zinc and mobilizable zinc, the latter being linked to unidentified non-protein ligands 22. Zn²⁺ is a cofactor for approximately 300 enzymes, enabling their catalytic activity, and it stabilizes the structure of nearly 2000 transcription factors. Furthermore, Zn²⁺ plays a central role in regulating critical cellular functions such as energy metabolism, gene expression, and genome integrity maintenance 23. However, abnormal Zn²⁺ accumulation induces irreversible cytotoxic effects. Vesicular exocytosis, zinc transport mediated by zinc transporters for entry or exit from the cell or organelle, and the binding or dissociation of MTs with zinc are the three primary sources of zinc signals. Given the absence of dedicated zinc storage systems, maintaining zinc homeostasis through regulated intake and excretion is essential. Two counteracting zinc transporter families primarily regulate zinc homeostasis. ZnT proteins, which mediate zinc efflux from the cytoplasm to organellar lumens or extracellular spaces, are encoded by the SLC30 family 24. Conversely, the SLC39 (ZIP) family mediates zinc influx from extracellular spaces or intracellular storage compartments into the cytoplasm, increasing intracellular zinc levels 25. MTs serve as primary intracellular zinc storage proteins, preventing zinc toxicity while maintaining zinc availability. MTF-1 contains six cysteine₂-histidine₂ zinc finger domains that mediate DNA binding. Consequently, in response to zinc signals, MTF-1 regulates zinc-responsive genes, including ZnT1, ZnT2, and MT. These MTs, characterized by high zinc affinity, function as primary intracellular zinc-binding proteins 26. Thiol oxidation to disulfide bonds triggers Zn²⁺ release. Disulfide reduction restores zinc-binding capacity. Cytosolic-free zinc concentrations are tightly regulated, with most intracellular zinc sequestered in organelles (ER, Golgi, mitochondria) comprising the cellular zinc store. Emerging evidence indicates zinc's dual roles as a neurotransmitter in intercellular communication and as an intracellular second messenger, transducing extracellular signals through diverse signaling pathways 27. Intracellular zinc signaling, including early (EZS) and late (LZS) phases, is activated by downstream pathways that mediate biological functions, including inflammatory signaling, and play key roles in the initiation, progression, and extinction of inflammatory responses to maintain immune homeostasis (Figure 1A).

### Regulation of zinc ions in tumorigenesis and signal transduction mechanisms

The role of Zn²⁺ in cancer depends on the type of cancer. Elevated Zn²⁺ levels in certain cancers correlate with reduced telomere attrition, a major contributor to chromosomal instability. Additionally, Zn appears to be protective in maintaining DNA 28. The regulation of zinc-associated proteins also varies by type and period of cancer. Dysregulated zinc homeostasis, exceeding cellular regulatory capacity, promotes tumorigenesis by supporting malignant bioenergetic and biosynthetic demands through mechanisms including zinc transporter dysregulation and MT binding protein alterations 29. Zinc-mediated activation of tumorigenic MAPK pathways, particularly ERK and JNK signaling, plays crucial roles in cancer development. These serine/threonine kinases (ERK, JNK) regulate tumorigenic processes including proliferation, differentiation, and apoptosis 30, 31. The Snail transcriptional repressor is activated in late zinc signaling via STAT3-mediated ZIP6 expression, which facilitates the epithelial-mesenchymal transition (EMT) throughout development and tumor metastasis. Similarly, via the PI3K/Akt signaling pathway, ZIP4 promotes migration and invasion linked to EMT in nasopharyngeal cancer. Furthermore, in ovarian cancer, high ZIP13 levels activate the Src/FAK pathway, which causes pro-metastatic genes to be upregulated and tumor suppressor genes to be downregulated 29, 32. The maintenance of cancer cells' invasiveness necessitates zinc stimulation of oncogenic pathways. For example, messenger RNA analysis of ZIP and ZnT proteins in human pancreatic adenocarcinoma shows that all ZIP proteins are downregulated, except ZIP4, which is increased 33. ZIP4 overexpression increases cell proliferation by activating the CREB pathway and promoting the silencing of miR-373 target genes, including PHLPP2, an inhibitor of pancreatic cancer cell proliferation, leading to cell proliferation and tumor progression. ZIP4 also decreases the expression of tight junction proteins, including ZO-1 and claudin-1, thereby contributing to the progression of tumors 34. Numerous studies have confirmed that dysregulation of zinc transporter proteins causes changes in different signaling pathways, which in turn promote cancer growth, in addition to affecting cell proliferation and death. Interestingly, it has been discovered that the lysosomal cation channel MCOLN1 plays a critical role in mediating zinc influx into the cytoplasm, which in turn fine-tunes oncogenic autophagy in malignant cells 32, 35, 36. Furthermore, it has been demonstrated that changes in zinc homeostasis modify the tumor immunological microenvironment, which has a major impact on cancer development 37. In summary, the link between zinc homeostasis and cancer requires further research to fully understand how its disruption leads to carcinogenesis.

### Regulation of apoptosis and pyroptosis in tumor cells by zinc ions

Apoptosis is a programmed cell death process that maintains tissue homeostasis by activating caspase proteases. In contrast, pyroptosis is an inflammatory cell death mediated by gasdermin proteins, characterized by membrane pore formation, cell expansion, and content release, triggering inflammation. Zn²⁺, a key signaling molecule, regulates both processes. It modulates apoptosis through mitochondrial function and caspase activation and induces pyroptosis by activating inflammasomes and ROS generation. Its dual role in these cell death mechanisms makes it a critical target for studying tumor cell fate 38 (Figure 1B). The mechanisms of zinc-based nanomaterials for tumor treatment and their combined therapeutic applications are summarized in Table 1.

#### Multiple pathways mechanism of zinc ion-induced apoptosis in tumor cells

Zn^2+^ overload induces reactive ROS production in tumor cell mitochondria, and its mechanism is mainly realized through two pathways. On the one hand, Zn²⁺ enters the mitochondria via ZnT2 and the mitochondrial calcium unidirectional transporter, inhibiting the function of the electron transport chain and leading to the accumulation of mitochondrial O₂^-^ and a decrease in mitochondrial membrane potential (MMP) 39. On the other hand, NADPH oxidase 1 causes damage to mitochondria by oxidizing NADPH to produce O₂^-^_,_ which is further converted into H₂O₂ and subsequently into hydroxyl radical (·OH). Damaged mitochondria release cytochrome c (Cyt-c), which interacts with apoptosis-activating factor 1 to form apoptotic vesicles. These vesicles activate caspase-9, leading to the subsequent activation of caspase-3, and ultimately initiating the process of apoptosis. In addition, Zn²⁺ treatment also leads to the down-regulation of caspase-8 expression, which may be mechanistically related to the exogenous apoptosis pathway mediated by tumor necrosis factor-α (TNF-α). Caspase-8 can directly cleave and activate caspase-3, which subsequently induces cell apoptosis. Meanwhile, MMP decline and Cyt-c release due to mitochondrial injury further activate caspase-9 and caspase-3, ultimately triggering endogenous mitochondria-mediated apoptosis 40.

Zn-induced ROS further activates the TBK1-IRF3 signaling cascade and the NF-κB signaling pathway. The activation of the NF-κB pathway, in turn, stimulates the production of inflammatory factors, including TNF-α, IL-6, and IL-1β 41. These inflammatory factors not only activate immune cells but may also indirectly promote apoptosis by affecting the cellular microenvironment 42. In addition, Zn²⁺ plays a key role in activating the STING pathway. Zn²⁺ overload induces mitochondrial damage, leading to the release of mitochondrial DNA (mtDNA) into the cytoplasm. The released mtDNA is then recognized by cGAS as an endogenous damage-associated molecular pattern. At the same time, ROS generated by Zn²⁺ inhibition of the mitochondrial electron transport chain can enhance the binding capacity of cGAS to DNA through oxidative modification, and the two together contribute to the generation of the second messenger, cGAMP, catalyzed by cGAS 43. cGAMP binds to and activates STING proteins on the surface of the endoplasmic reticulum, which recruits the key kinase TBK1, phosphorylates the transcription factor IRF3, and activates the STING protein, which recruits the key kinase TBK1. IRF3 phosphorylates, and activated IRF3 dimerizes and enters the nucleus, inducing transcription of type I interferons (e.g., IFN-β) and proinflammatory factors (e.g., IL-6), which not only activate immune cells but may also indirectly promote apoptosis 44. Moreover, Zn²⁺ ions may amplify immune- and apoptosis-related signals by enhancing cGAS activity and directly activating the STING pathway 45.

#### Multiple pathways mechanism of zinc ion-induced pyroptosis in tumor cells

Zn-induced generation of ROS activates the NF-κB signaling pathway on the one hand and leads to mitochondrial damage on the other. Together, these two act as initiating and activating signals for NLRP3 inflammatory vesicles. The activated NLRP3 inflammasome further activates caspase-1, which cuts gasdermin D (GSDMD) to generate its N-terminal fragment GSDMD-N. The GSDMD-N fragment forms a pore in the cell membrane, leading to the release of cellular contents (e.g., IL-1β and IL-18), which in turn trigger cell death. In gasdermin E (GSDME)-expressing cells, Zn²⁺ treatment activates caspase-3, and the activated caspase-3 specifically cleaves GSDME to generate GSDME-N fragments 46. Similar to GSDMD-N, GSDME-N fragments can form pores in the cell membrane, destroying the integrity of the cell membrane and inducing cell death. In addition, Zn-induced ROS damages mitochondria, leading to the release of mitochondrial DNA (mtDNA) into the cytoplasm. The released mtDNA induces the formation of AIM2 inflammatory vesicles, and activates caspase-1, which cleaves GSDMD, generating GSDMD-N fragments, and ultimately induces cell death. During this process, the released inflammatory factors such as IL-1β and IL-18 not only recruit immune cells and enhance the immune response, but also further exacerbate the occurrence of cell death. Zn²⁺ regulates the expression of Bcl-2 family proteins, up-regulating the expression of pro-apoptotic Bax and other pro-survival pathways, down-regulating the expression of anti-apoptotic Bcl-XL and Bcl-2, and altering the permeability of mitochondrial membranes. Different concentrations of Zn²⁺ show biphasic effects on the regulation of signaling pathways: low concentrations usually exert anti-apoptotic effects such as inhibition of caspases and activation of pro-survival pathways (e.g., PI3K/Akt), whereas high concentrations can induce oxidative stress, activate pro-apoptotic pathways (e.g., JNK/p38 MAPK), or cause mitochondrial dysfunction 47. Zn^2+^ plays various roles in immunomodulation. Zn^2+^ can inhibit the binding of protein tyrosine phosphatase 1 (SHP-1) to T-cell antigen receptors by increasing intracellular concentration, maintaining T-cell activation signaling and calcium inward flow, and up-regulate the expression of the co-stimulatory molecules, CD80 and CD86, which can promote the antigen presentation and the maturation of dendritic cells. In addition, Zn^2+^ participates in the phosphorylation of IL-1 receptor-associated kinase and MyD88 signaling pathway, and promotes the secretion of pro-inflammatory cytokines such as IFN-γ and TNF-α, and chemokines such as CXCL-5 and CXCL-10, thus regulating immune response through multiple pathways 48.

In summary, Zn²⁺ induces apoptosis and pyroptosis in tumor cells through the aforementioned multiple pathways. The inflammatory factors produced during this process, such as IFN-β, TNF-α, IL-6, and IL-1β, play important roles in immune responses, collectively triggering systemic anti-tumor immunity and inhibiting tumor growth. Zn²⁺ exhibits significant anti-tumor potential by inducing pyroptosis and immune activation, but single therapies are often difficult to overcome tumor complexity and drug resistance. Therefore, combining Zn²⁺ with other therapeutic agents has become an important research direction.

## Zinc Ions in Combination with Other Therapies

**Figure 2:**
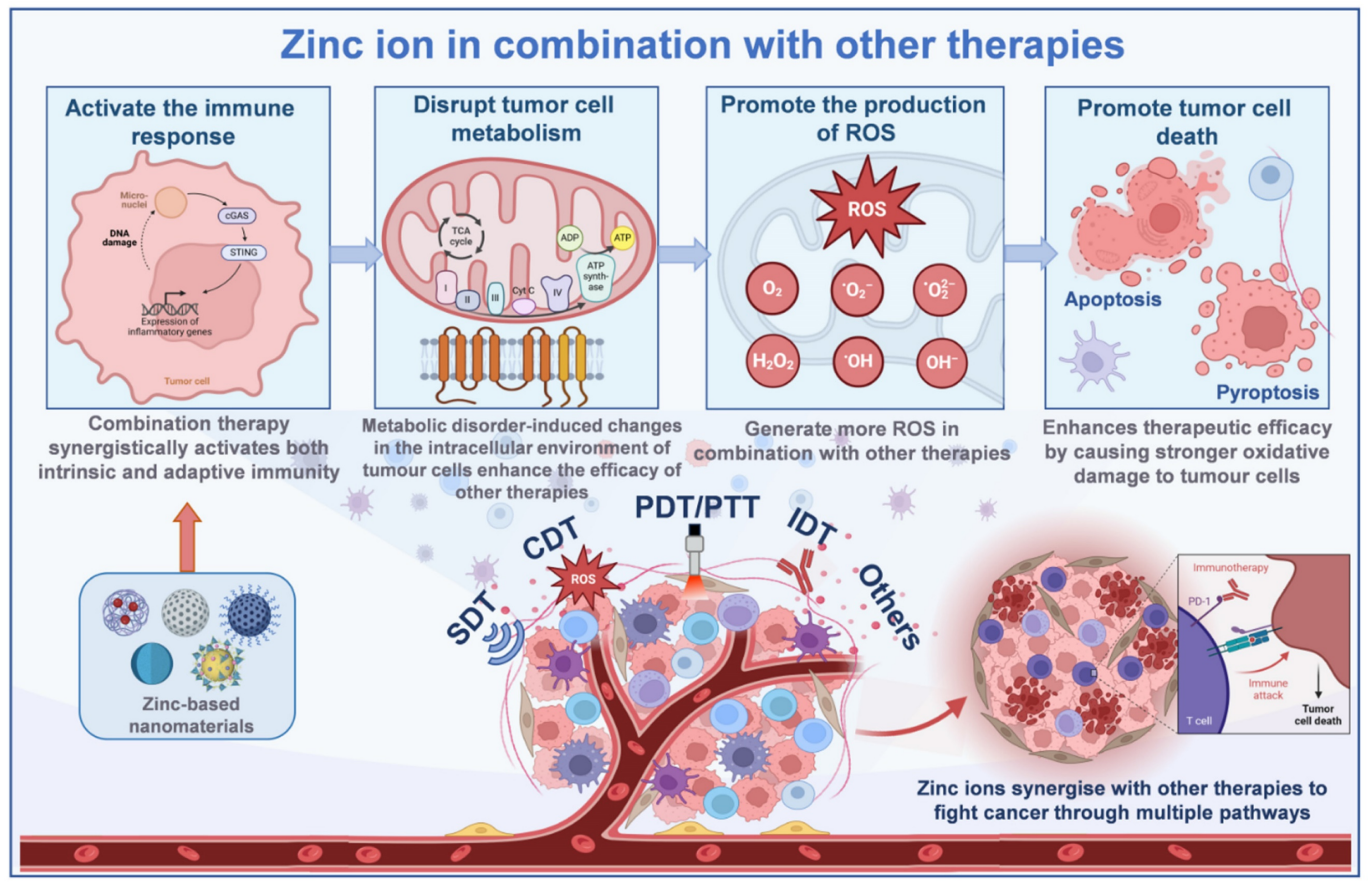
When combined with photodynamic, acoustic, chemodynamic, and immunotherapeutic agents, Zn²⁺ enhances ROS generation by inhibiting the mitochondrial electron transport chain in photo-/acousticodynamic therapy, synergistically promotes reactive oxygen species (ROS) generation from the Fenton reaction with chemodynamic reagents, and induces immunogenic cell death to activate the anti-tumor immune response, providing a multidimensional strategy for combined tumor therapy. Created with BioRender.com. (http://biorender.com)

As introduced earlier, Zn²⁺ as a metal ion induces tumor cell death with its unique advantages. However, in the face of the complex microenvironment of tumors, single therapies are prone to make tumor cells resistant to treatment. Therefore, a combination of mechanisms is usually used to enhance the therapeutic effect during research (Figure 2). When used alone, both acoustic and photodynamic therapies have a limited ability to generate ROS 49. The addition of Zn²⁺ triggers a mitochondrial ROS burst by inhibiting the mitochondrial electron transport chain: in acoustic kinetic therapy, the ultrasound-triggered ROS generated by the photosensitizer binds to Zn-induced ROS; in photodynamic therapy, the light-activated ROS generation works synergistically with the Zn-induced ROS to dramatically increase the level of ROS in tumor cells. Specifically, Zn-based nanomaterials (e.g., ZnO₂) release Zn²⁺ and H₂O₂ in acidic tumor microenvironments, and H₂O₂ serves as a substrate for the photosensitizers (e.g., Ce6) in PDT, generating more ·OH and ¹O₂ through Fenton or photocatalytic reactions, while Zn²⁺ inhibits the mitochondrial electron transport chain, thereby increasing electron leakage to oxygen molecules, further promoting O₂- accumulation and forming a “Zn²⁺-ROS” positive feedback loop. Elevated levels of ROS oxidize intracellular lipids, proteins, DNA, and other biomolecules, disrupting the function of cellular structure and greatly increasing the anticancer effect, leading to tumor cell necrosis or apoptosis 46. In addition, Zn²⁺ interferes with the energy metabolism of tumor cells, blocking key processes such as NAD⁺ synthesis and glycolysis, so that the energy supply of tumor cells is impeded, and tumor cells in a state of metabolic stress are more sensitive to acoustic/photodynamic therapy; at the same time, Zn²⁺ reduces intracellular ATP by inhibiting key enzymes of glycolysis (e.g., LDH). Additionally, O₂ produced from the decomposition of ZnO₂ relieves tumor hypoxia, overcoming the limitations of the therapeutic efficacy of PDT in the hypoxic region. Metabolic disorders induce changes in intracellular pH and redox potential in tumor cells, further promoting the production of more ROS by PDT and SDT, enhancing the efficacy of the combination therapy 50. Certain Zn-based nanomaterials (e.g., ZnO and ZIF-8), which combine acoustic and photosensitizing properties, can effectively generate ROS and induce immunogenic cell death (ICD) to activate anti-tumor immune responses: Zn²⁺ activates the cGAS/STING pathway through ROS-mediated mtDNA release, promotes IFN-β secretion, and interacts with damage-associated molecular patterns (DAMPs) released by PDT. DAMPs released by PDT synergistically enhance dendritic cell antigen presentation, while Zn²⁺ stimulates the immune system to promote immune cell infiltration. The combination of the two therapies can synergistically activate both intrinsic and adaptive immunity, further increasing the efficacy of the treatment and significantly reducing the development of drug resistance 51.

Chemodynamic therapy (CDT) primarily relies on the Fenton or Fenton-like reaction, which catalyzes the conversion of H₂O₂ into highly toxic·OH to destroy tumor cells. However, the effectiveness of this therapy is constrained by the low concentration of H₂O₂ in the tumor microenvironment 52. The introduction of Zn²⁺ can elevate intracellular oxidative stress by inhibiting the mitochondrial electron transport chain and promoting the generation of large amounts of ROS within mitochondria. In addition, Zn^2+^ can enhance the catalytic activity of Fenton-like systems by synergistically interacting with metastable metals (e.g., Fe, Cu) or carbon-based materials to promote the conversion of H_2_O_2_ to ·OH and increase the efficiency of hydroxyl radical generation, which synergistically leads to the accumulation of large amounts of ROS in tumor cells, resulting in stronger oxidative damage and inducing apoptosis 53. Zn²⁺ depletes intracellular GSH in tumor cells, disrupting redox homeostasis and enhancing oxidative stress by either directly binding to GSH to form a complex or indirectly catalyzing ROS generation to prompt GSH oxidation. This not only promotes ROS accumulation in the CDT but also optimizes the tumor microenvironment to increase CDT efficiency and enhance synergistic effects with other therapeutic strategies.

When acting alone, Zn²⁺ can induce multiple forms of tumor cell death, including apoptosis and pyroptosis. In immunotherapy, tumor-associated antigens released during immunogenic cell death stimulate the adaptive immune response. Zn²⁺ combined with immunotherapy induces tumor cells to die in a more immunogenic manner, releasing more tumor-associated antigens. These antigens are taken up, processed, and presented by antigen-presenting cells, which activate T-lymphocytes to generate specific anti-tumor immune responses and enhance the targeting and killing ability of immunotherapy on tumor cells 47. Zn²⁺ can also neutralize the acidity of tumor cell lysosomes, which interferes with autophagy and increases the vulnerability of tumor cells to immune system recognition and assault. This modulation of the tumor microenvironment establishes more favorable conditions for immunotherapy, enhancing the infiltration and activity of immune cells within tumor tissues. Tumor cells frequently evade immune surveillance and attack through various mechanisms, including the overexpression of immune checkpoint proteins. Zinc ion-based combination immunotherapy can decrease the expression of these immune checkpoint proteins in tumor cells, thereby reducing immune escape 54. On the one hand, Zn²⁺ regulates the process of tumor cell metabolism and microenvironment, which can affect the expression regulation of immune checkpoint proteins; on the other hand, immune checkpoint inhibitors used in immunotherapy synergize with Zn²⁺ to restore the immune system's function of recognizing and killing tumor cells, and improve the effectiveness of the combination therapy.

## The Application of Zinc Nanomaterials in Cancer Therapy

Zinc-based nanomaterials have emerged as promising therapeutic agents based on the unique role of Zn²⁺ in tumor metabolism and immunomodulation. These materials have greatly improved the efficacy of tumor therapy by targeting Zn²⁺ release, inducing oxidative stress, and activating immune responses. This section will systematically review the progress of zinc-based nanomaterials (e.g., ZnO₂, ZnO, ZIF-8, and ZnS) in different cancer therapeutic modalities, as well as their innovative applications in multimodal therapeutic and combinatorial strategies. Information regarding the mechanism of zinc-based nanomaterials for the treatment of tumors and their combined therapeutic applications are shown in Table 2.

### Application of ZnO_2_ in tumor therapy

Nanozyme-mediated chemodynamic therapy has become a promising strategy due to its tumor specificity and controllable catalytic activity. However, a major challenge persists due to the limited therapeutic efficacy caused by insufficient H₂O₂ levels in the TME 55. Currently, the most effective method is that metal peroxides characterized by metal ions and peroxy groups utilize the acidic pH of the TME and trigger the in-situ production of H₂O₂, enhancing the oxidative stress of tumor cells. Additionally, metal ions (e.g., Zn²⁺/Fe²⁺) released from peroxides accumulate in tumor cells, inducing ionic overload and amplifying oxidative stress in the TME, thereby enhancing therapeutic efficacy 56. ZnO₂ NPs induce tumor-specific Zn²⁺ accumulation and overload via endocytosis, triggering apoptotic signaling cascades. In the TME, ZnO₂ decomposes into H₂O₂ and Zn²⁺, disrupting zinc homeostasis while promoting endogenous ROS generation and oxidative stress 57. Sun *et al.* designed the ZnO_2_@Pt platform that generated ROS at the tumor site, induced apoptosis, and inhibited glycolysis, achieving 89.7% tumor growth inhibition 58. Therefore, the combination of ZnO₂ and CDT offers a highly tumor-specific therapeutic strategy that can minimize harm to normal cells while dramatically enhancing treatment selectivity.

**Figure 3:**
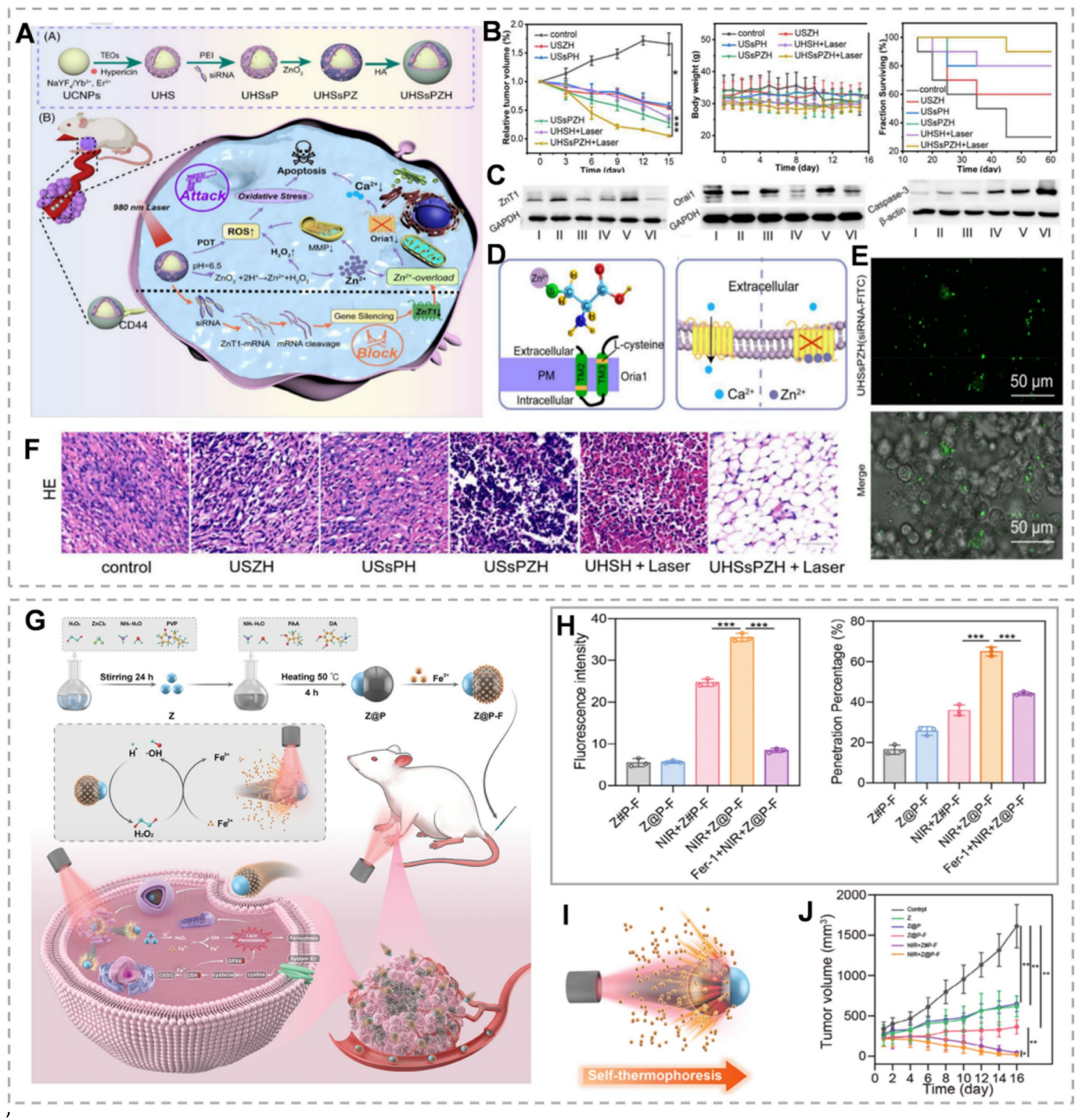
(A) Synthesis and anti-tumor mechanism of UHSsPZH nanoparticles. (B) Tumor growth, weight, and survival under different conditions. (C) Protein blotting of ZnT1, Orail, and Caspase-3 after different treatments. (D) Schematic diagram of Zn²⁺ chelation and the mechanism influencing Ca²⁺ transport. (E) Verification that siRNA is efficiently delivered to tumor cells. (F) Hematoxylin and eosin (H&E) staining in different groups to verify the treatment effect. (G) Schematic of self-thermophoretic nanomotors. (H) The permeability of cross-sections and the fluorescence intensity of tumor spheroids following different treatments. (I) NIR light-driven nanoparticles. (J) Tumor volume under different conditions. **(A-F)** Reproduced with permission from 61, copyright 2023, Wiley-VCH GmbH.** (G-H)** Reproduced with permission from 68, copyright 2024, Wiley-VCH GmbH.

As mentioned above, ZnT1, the sole plasma membrane Zn²⁺ efflux transporter, plays a critical role in maintaining cellular zinc homeostasis. ZnT1 suppression significantly enhances intracellular Zn²⁺ accumulation and overload 59. Small interfering RNA (siRNA) exhibits high specificity for target gene silencing post-transfection. siRNA-mediated gene silencing has emerged as a promising therapeutic strategy for cancer and other diseases 60. Therefore, inhibition of ZNT1 expression by siRNA could be an effective method to promote intracellular Zn²⁺ accumulation in tumor cells. For example, Shi *et al.* 61 developed the UHSsPZH NPs composite nanoparticles. The surface of synthesized UCNPs was modified with chrysoidine and mesoporous silica, followed by adsorption of siRNA, then encapsulation of ZnO₂, and modification of hyaluronic acid. This dual strategy combines Zn²⁺ efflux blockade through gene silencing with tumor-targeted ZnO₂ delivery. Post-endocytosis, intense green fluorescence from UHSsPZH NPs and siRNA-FITC in 4T1 cells confirmed effective siRNA delivery and ZnT1 silencing (Figure 3E). Additionally, Orai1, a calcium channel overexpressed in tumors that promotes Ca²⁺ oscillations and tumor cell proliferation, was significantly downregulated in the USsPZH and UHSsPZH + Laser groups, highlighting Zn²⁺ overload-mediated disruption of calcium homeostasis (Figure 3C). This Zn²-mediated suppression of Orai1 not only disrupted calcium homeostasis but also triggered caspase-dependent apoptosis. Importantly, the UHSsPZH + laser treatment demonstrated superior therapeutic efficacy, as evidenced by significantly prolonged survival in tumor-bearing animals (Figure 3B and F).

As previously discussed, elevated Zn²⁺ levels activate the p53 signaling pathway. The tumor suppressor p53 regulates cancer cell death by transcriptionally activating proapoptotic genes, including Bax. However, wild-type p53 (WTp53) levels are significantly reduced or undetectable in many human cancers 69. Therefore, simultaneously promoting Mutp53 degradation and WTp53 stabilization represents an effective anticancer strategy. Recent studies demonstrate that Zn-based nanomaterials induce substantial oxidative stress through mitochondrial ETC inhibition 70, 71. Importantly, elevated intracellular Zn²⁺ and ROS selectively degrade Mutp53 (both contact and conformational mutants) via the ubiquitin-proteasome system (UPS)-mediated degradation, while sparing WTp53 21. Furthermore, Mn²-mediated ATM-p53 pathway activation enhances WTp53 accumulation and stabilization. Mn²⁺ exposure activates ATM through autophosphorylation and phosphorylation of downstream targets (CHK2[T68], H2AX[S139]), leading to p53 phosphorylation 72. Chen's team demonstrated that Mn²⁺-ZnO₂ synergistically enhanced ROS generation, promoting Mutp53 degradation while activating WTp53 for cancer therapy 73. However, ZnCl₂ at equivalent Zn²⁺ concentrations showed limited Mutp53 degradation efficacy, likely due to poor membrane permeability. On the other hand, Zn²⁺ can enhance manganese-catalyzed ·OH synthesis by increasing endogenous reactive oxygen species generation, and thus the secretion of ATP, to express tumor-associated antigens (TAAs) and DAMPs, and to induce an ICD effect 74. In addition, manganese ions improve the ability of cGAS to bind to double-stranded DNA, triggering the cGAS/STING pathway in antigen-presenting cells. This activation strengthens tumor-specific T-cell responses and boosts the production of pro-inflammatory cytokines and chemokines 75. Zhou *et al.* 20 developed TME-responsive manganese-rich ZnO₂ nanoparticles (MOMPs) by doping Mn^2+^ in ZnO₂NPs, which could synergistically enhance anti-tumor immunotherapy through ICD induction and STING pathway activation. MONPs alleviate TME immunosuppression by reducing regulatory T cells and polarizing M2 to M1 macrophages, creating an immunologically supportive environment for adaptive immune responses. Thus, combining ZnO₂ with Fenton reagents or promoting Fenton-like reactions can synergistically enhance ZnO₂-mediated anti-tumor efficacy by amplifying ROS generation and modulating the tumor microenvironment.

**Figure 4:**
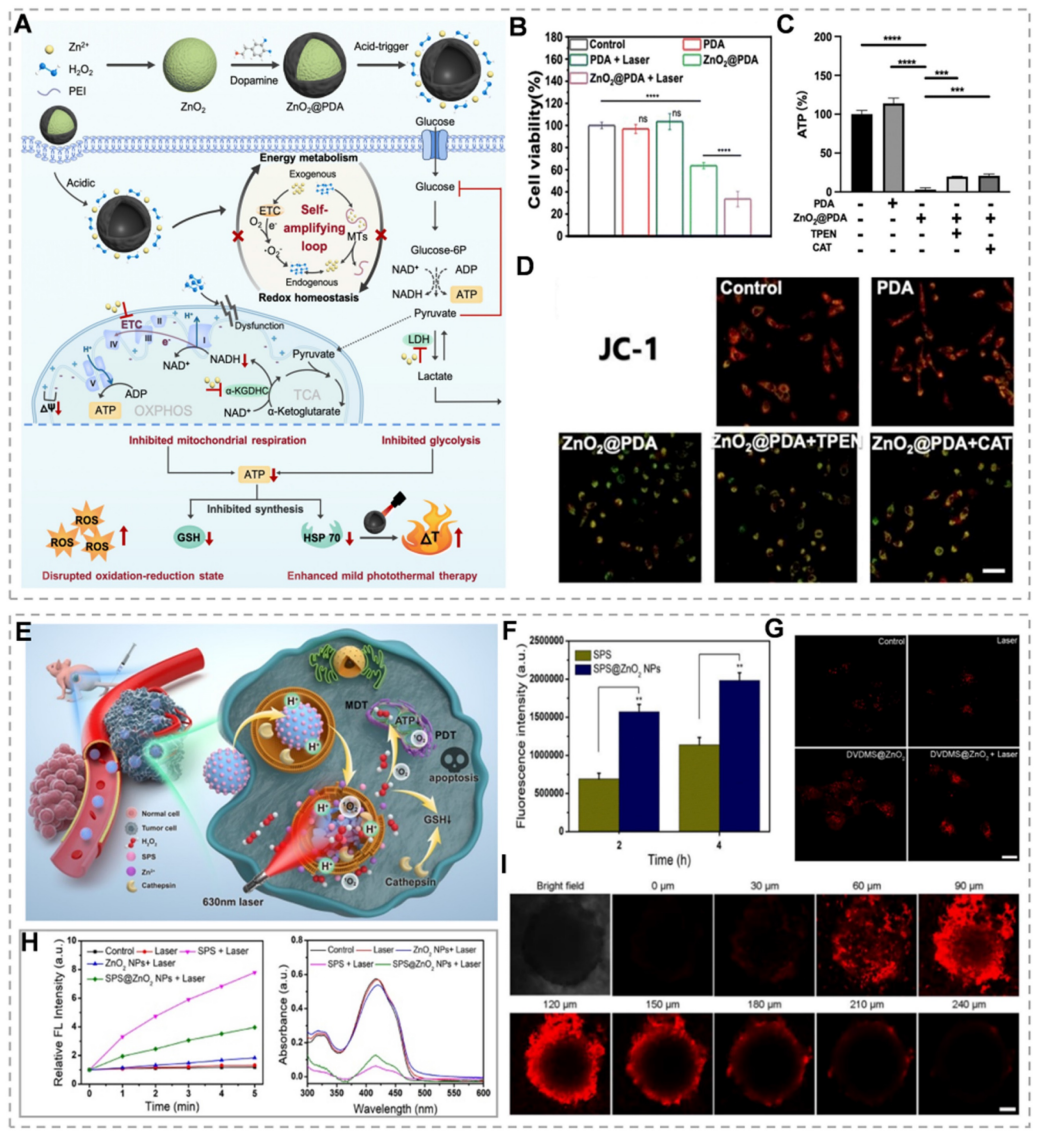
(A) Schematic synthesis and mechanism of ZnO₂@PDA nanoparticles. (B) Cell viability of different treatments. (C) ATP levels in cells with different treatments. (D) JC-1 assay for mitochondrial membrane potential in HeLa cells following different treatments. (E) Schematic diagram of the mechanism of action of SPS@ZnO₂ NPs. (F) Confocal microscope images of 4T1 cells under different conditions. (G) Cathespin B release from lysosomes to cytosol induced by SPS@ZnO₂ NPs in 4T1 cells. (H) Oxygen generation in single line state for different groups. (I) Z-axis scanning images of tumors after treatment with SPS@ZnO₂ NPs. **(A-E)** Reproduced with permission from 78, copyright 2024, Royal Society of Chemistry. **(F-I)** Reproduced with permission from 80. copyright 2021, American Chemical Society.

ZnO₂ concentration determines cancer cell death modalities. High ZnO₂ concentrations generate elevated H₂O₂ levels, inducing pyroptotic cell death. Elevated H₂O₂ activates the NLRP3 inflammasome and caspase-1, resulting in GSDMD cleavage and IL-1β maturation. The GSDMD-N forms plasma membrane pores, triggering cytokine release, immune activation, and pyroptotic cell lysis. In contrast, low ZnO₂ concentrations generate sublethal H₂O₂ levels, inducing apoptosis without significant cell lysis. Therefore, pyroptosis elicits stronger immune activation and antitumor effects compared to apoptosis 76. Zn²⁺ promotes endogenous and exogenous H₂O₂ generation through multiple pathways, establishing a Zn²-H₂O₂ self-amplifying loop. Zn²⁺ inhibits key metabolic enzymes in glycolysis and mitochondrial respiration, blocking ATP production. PTT achieves tumor cell ablation at lower temperatures, minimizing off-target thermal damage and improving treatment tolerability. However, heat shock proteins (HSPs) repair heat-damaged proteins, conferring thermoresistance and reducing therapeutic efficacy 77. Because HSPs' biosynthesis and activity are dependent on ATP, Zn²-mediated ATP depletion may enhance PTT efficacy by inhibiting the cytoprotective effects of HSPs. Based on this, Qiao's team prepared polydopamine-shell-encapsulated ZnO₂ nanoparticles (ZnO₂@PDA NPs), aiming to accurately target the tumor energy metabolism-redox circuit through the synergistic effects of Zn²⁺ and hydrogen peroxide (Figure 4A). ZnO₂@PDA disrupts cellular energy metabolism, evidenced by decreased NADH and ATP levels (Figure 4C). JC-1 probe analysis revealed mitochondrial depolarization in both ZnO₂@PDA and ZnO₂@PDA + laser groups, indicating mitochondrial dysfunction (Figure 4D). ZnO₂@PDA with 808 nm laser irradiation showed enhanced HeLa cell toxicity compared to ZnO₂@PDA alone (Figure 4B). This suggests that the inhibitory effect of ZnO₂@PDA on ATP production amplifies the effect of PDA-mediated mild PTT 78. Alternatively, H₂O₂ exerts tumoricidal effects through molecular dynamic therapy (MDT). MDT is an emerging therapeutic strategy that utilizes endogenous H₂O₂ to deplete intracellular GSH, which can neutralize the effect of GSH on PDT-produced ^1^O_2_ and thus enhance the efficacy of PDT 79. For example, Zhang *et al.* 80 constructed a biodegradable tumor microenvironment-responsive nanoplatform, SPS@ZnO₂ NPs, by loading sodium porphyrin photosensitizer (SPS) with ZnO₂ NPs as a carrier. On the one hand, it enhances the effect of MDT by depleting GSH, and on the other hand, it prompts the production of ROS by SPS to initiate PDT under the irradiation of a 630 nm laser, which realizes the synergistic treatment of MDT/PDT. Under 630 nm laser irradiation, these nanoparticles generate cytotoxic singlet oxygen (¹O₂) while enhancing photosensitizer uptake and intracellular penetration (Figure 4F, H and I). H₂O₂ generation and cathepsin B release deplete intracellular GSH, potentiating PDT efficacy (Figure 4G). This expands the therapeutic potential of Zn²⁺ peroxide-based materials in oncology.

ZnO₂ nanomaterials show great potential in cancer therapy, capable of selectively targeting cancer cells and reducing damage to healthy tissues. However, their application still faces many challenges: Zn²⁺ concentration is difficult to precisely regulate, hydrogen peroxide supply is insufficient and the reaction efficiency is low, tumor biological barriers limit the penetration of the materials, and photodynamic combination therapy has limited effects in deep-seated tumors. Specifically, high or low Zn²⁺ concentration leads to normal tissue toxicity or insufficient immune activation, respectively; low hydrogen peroxide concentration in tumors limits ROS generation, and the complexity and heterogeneity of the tumor microenvironment further hinder therapeutic efficacy. To overcome these challenges, precisely controlled-release systems can be constructed and tumor microenvironment-responsive carriers can be designed to achieve accurate release of Zn²⁺ and hydrogen peroxide. Meanwhile, optimizing the response system to improve the efficiency of ROS generation and combining it with other therapeutic tools such as immunotherapy, is expected to enhance the synergistic therapeutic effect. In addition, the development of nanomaterials with active targeting function to break through biological barriers and the adoption of near-infrared two-region photodynamic technology to increase the depth of light penetration will provide new ideas for the application of ZnO₂ in deep tumor therapy and promote its further development in the field of cancer therapy.

### Application of ZnO in tumor therapy

Zinc oxide nanoparticles (ZnO NPs) serve as versatile agents in cancer therapy, functioning as chemosensitizers to enhance the efficacy of pharmaceuticals and as photosensitizers to generate ROS under light irradiation, thereby inducing apoptosis in tumor cells. Beyond these functions, ZnO NPs serve as targeted drug delivery vehicles and cancer diagnostic biosensors. Through oxidative stress modulation, cell cycle regulation, and mitochondrial targeting, ZnO NPs demonstrate cancer cell-selective cytotoxicity, offering significant therapeutic advantages with reduced side effects 81, 82. ZnO NPs enter lysosomes through autophagy and dissolve to release Zn²⁺, leading to cytotoxicity. On one hand, Zn²⁺ can impair mitochondria and lysosomes, further disrupting the negative feedback loop between ROS and mitochondrial autophagy. This results in mitochondrial dysfunction, ROS overproduction, and cell death. Additionally, released Zn²⁺ downregulates β-catenin via the HIF-1α/BNIP3/LC3B mitophagy pathway, suppressing tumor metastasis 83. Table 3 summarizes ZnO's cancer therapeutic applications, mechanisms, effects, and research progress.

**Figure 5:**
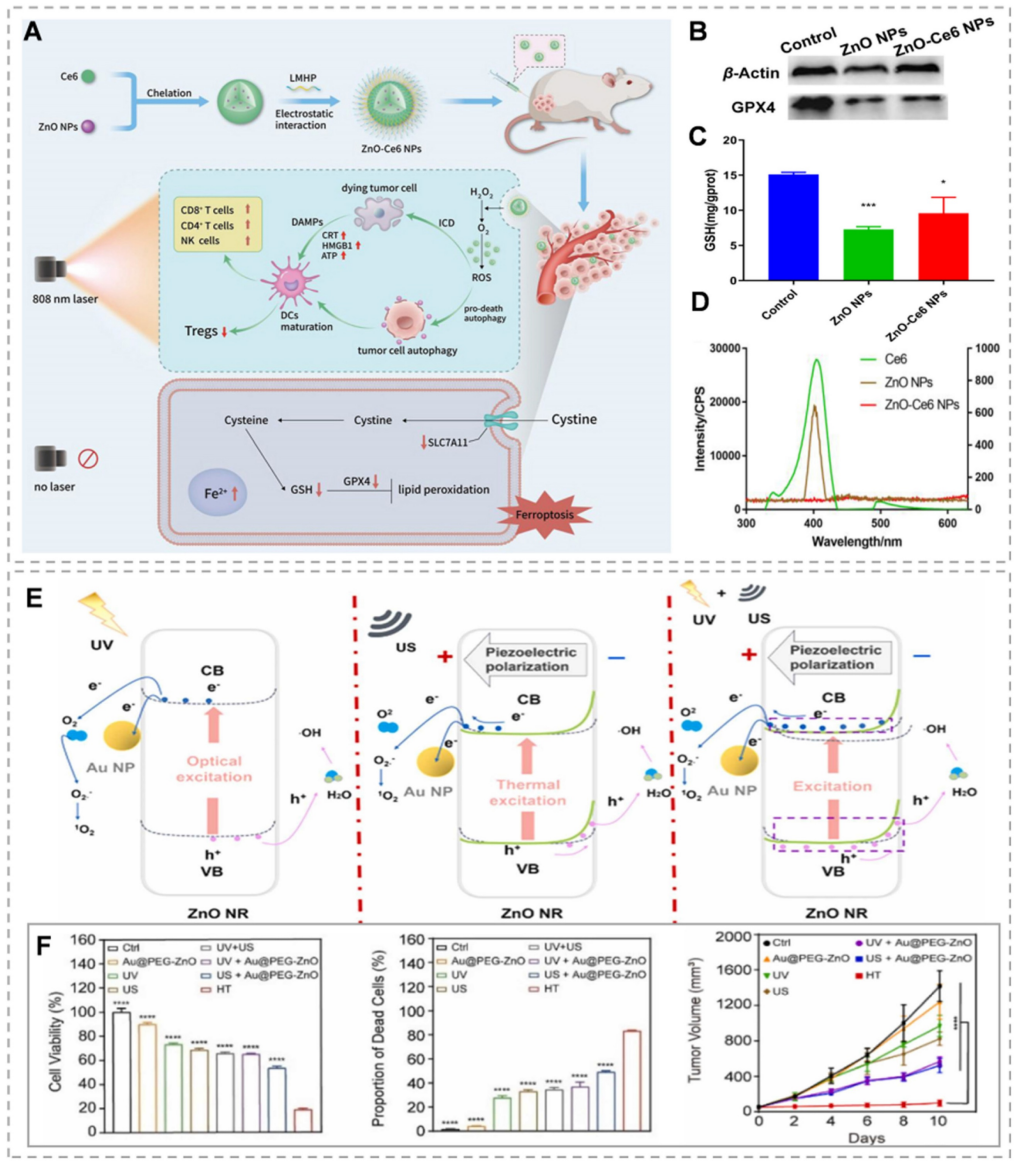
(A) Preparation of ZnO-Ce6 NPs and elaboration of their mechanism of action. (B) Detection of GPX4 protein expression levels by Western blotting. (C) The level of GSH was detected using the DTNB colorimetric method. (D) Demonstration of excitation wavelength variation in ZnO-Ce6 nanoparticles. (E) Mechanism map of ROS generation by Au@PEG-ZnO NPs under different conditions. (F) The therapeutic effects of Au@PEG-ZnO *in vitro* and *in vivo*. **(A-D)** Reproduced with permission from 87, copyright 2024, Elsevier B.V. **(E-F)** Reproduced with permission from 96, copyright 2024, Elsevier B.V.

ZnO, as a semiconductor with a band gap of 3.37 eV and an exciton binding energy of 60 meV at room temperature, initiates photocatalytic reactions when it absorbs photons with energy greater than its band gap. This process is driven by visible or UV light, which causes electrons to transition from the valence band to the conduction band, creating electron-hole pairs. These charge carriers can rapidly recombine or migrate to the nanoparticle surface to interact with surrounding molecules. The conduction band electrons act as reductants, reducing ambient oxygen to O_2_^-^, while the valence band holes, being potent oxidants, react with water to produce ·OH, H_2_O_2_, and protonated superoxide radicals (HO_2_·). These reactions initiate subsequent chain reactions involving a variety of ROS 84. The main challenges of PDT therapy include the restricted penetration depth of light in tumor tissue and the inadequate production and accumulation of ROS in hypoxic tumors 85. ZnO can emit 401 nm visible light when exposed to 808 nm near-infrared (NIR) light, thereby offering the potential to replace 401 nm visible light with 808 nm NIR light. By leveraging this property, ZnO can be combined with other photosensitizers to extend the wavelength range into the NIR region, thereby improving penetration 86. Wang *et al.* 87 developed zinc oxide-chlorin Ce6 nanoparticles (ZnO-Ce6 NPs) (Figure 5A), 808 nm laser irradiation of ZnO-Ce6 NPs triggered 401 nm visible light emission from ZnO NPs, stimulating Ce6 to induce photodynamic action (Figure 5D). In addition, ZnO-Ce6 NPs can enhance the decomposition of H₂O₂ to generate oxygen through the enzymatic activity of ZnO NPs, supplying ample oxygen for PDT, alleviating hypoxia, and normalizing tumor blood vessels. Even without light, ZnO-Ce6 NPs can induce cell death through ferroptosis (Figure 5B and C). However, the inadequate harnessing of solar energy by ZnO, attributed to its wide band gap, leads to diminished efficiency in photocatalytic hydrogen generation. In addition, the fundamental deficiency of UV light in penetrating deeply into tissues, given by its high absorption rate by the nearby biological context 88. The addition of noble metals has been shown to significantly improve the photocatalytic efficiency of ZnO. This enhanced photoelectrochemical activity is attributed to the efficient transfer of charges from the metal core to the conduction band of ZnO, which helps reduce the recombination of electrons and holes 89. Zhou *et al.* 90 investigated the construction of core-shell nanostructures (M@ZnO) by combining noble metal nanoparticles (Au, Au@Ag) with mesoporous ZnO in different shapes (spherical, rod-like). The thickness of the ZnO shell layer was regulated by the ascorbic acid-assisted method, and its visible-light-driven photoelectrochemical water decomposition performance was systematically investigated. The results show that the core-shell structure inhibits the photogenerated electron-cavity pair complexation and broadens the visible-light response range of ZnO through effective charge transfer from metal to ZnO. Among them, Au@Ag@ZnO exhibits higher photocurrent density than pure Au@ZnO due to a lower Schottky barrier and wider visible extinction range. In addition, the optimal ZnO shell layer thickness (e.g., 34 nm for Au nanorods@ZnO) exists for M@ZnO with different metal cores, which can optimize the photoelectrocatalytic activity. Although various approaches have been designed to merge plasmonic metals with ZnO, such as core-shell structures and coating techniques, the ROS yield of the composite systems remains limited. Traditional core-shell structures and encapsulation methods fail to effectively isolate the spatial distribution of high-energy electron-hole pairs, thus necessitating the consideration of other materials' involvement 91. Graphene offers a strong framework for anchoring finely dispersed metal or oxide nanoparticles. It acts as a highly conductive substrate, ensuring efficient electrical contact and facilitating electron transfer from the semiconductor's conduction band. This enhances charge separation efficiency and catalyzes ROS generation, thanks to its large surface area and high electron mobility 92. Dong's team constructed the Au@ZnO@GQDs/HA (AZGH) nano heterostructure, integrating GQDs into the AZ core-shell structure, and successfully used it to promote tumor clearance through ROS generation mechanisms. Comparative analysis with AZ nanoparticles indicates that the synthesized AZG nano-heterostructures enhance ROS production under near-infrared excitation. This improved efficiency in generating ^1^O_2_ and ·OH is attributed to the rapid separation of electron-hole pairs. Additionally, the photocatalytic activity contributes significantly to combination therapy, and the decorated HA can target 4T1 cells, increasing the concentration of AZGH breakdown in 4T1 cells 93. Utilizing the piezoelectric effect in ZnO to create an internal electric field and reduce the recombination rate of photo-generated carriers may improve PDT performance 94. By employing US-induced vibrations to generate piezoelectric polarization charges in ZnO nanostructures, catalytic processes can be modulated through the internal piezoelectric field. Considering its distinctive amalgamation of photo-induced, piezoelectric, and mechano-catalytic characteristics, ZnO possesses the capacity to trigger dual catalytic reactions or potentially initiate innovative synergistic catalytic mechanisms 95. Piezo-photodynamic therapy (PPDT) can dually activate the electron-hole pairs within nanocomposite materials through the application of both ultrasound and ultraviolet radiation. The internally generated piezoelectric field reduces electron-hole recombination, significantly boosting the production of ROS within tumor cells. The PPDT approach outperforms traditional PDT and SDT, showcasing synergistic therapeutic effects. Han *et al.* 96 designed and prepared Au@PEG-ZnO nanocomposites with ZnO NRs as the main body to reduce carrier complexation and enhance ROS generation by utilizing their piezoelectric and photocatalytic effects. The Au NPs further promote electron-hole separation by constructing metal-semiconductor heterojunctions. This dual activation mechanism enables electron-hole pair generation from the nanocomposite material through US and UV stimulation (Figure 5E). *In vivo* and *in vitro* experiments using a preclinical mouse lung cancer model show enhanced therapeutic results compared to standalone PDT and SDT treatments (Figure 5F).

**Figure 6:**
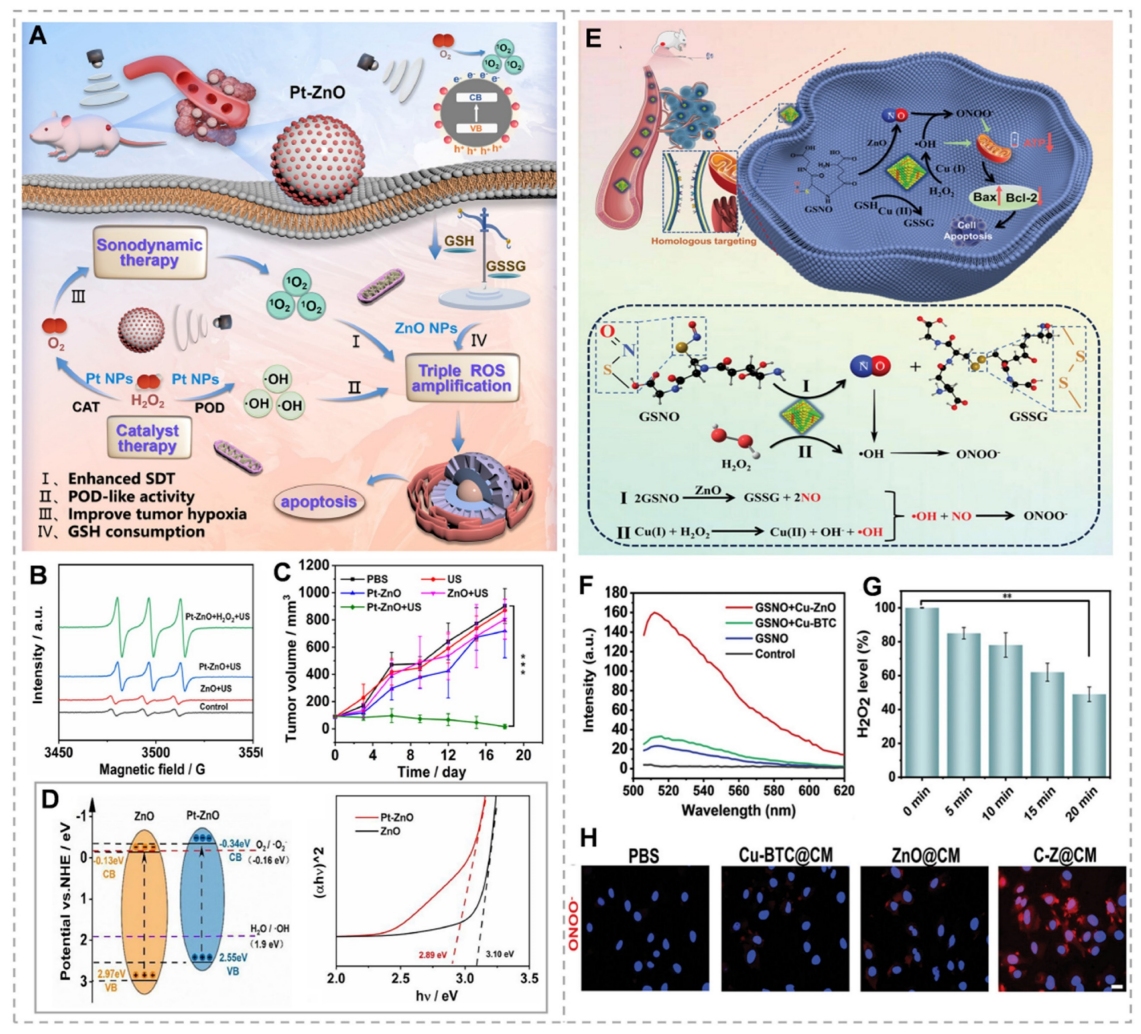
(A) Diagram of the mechanism of action of Pt-ZnO NPs. (B) Detection of ¹O₂ production of Pt-ZnO NPs in the presence of ultrasound. (C) Tumor growth in different treatments. (D) The heterogeneous structure of Pt-ZnO improves the separation efficiency of electrons (e⁻) and holes (h⁺) under ultrasound irradiation. (E) Schematic illustration of the multimodal antitumor effects of C-Z@CM. (F) Verify that Cu-ZnO produces NO. (G) The levels of H₂O₂ at different time points after treatment with Cu-ZnO. (H) Detection of intracellular ONOO⁻ levels using an ONOO⁻ probe. **(A-D)** Reproduced with permission from 99, copyright 2023, Elsevier B.V. **(E-H)** Reproduced with permission from 106, copyright 2024, Wiley-VCH GmbH.

Relative to the light employed in photodynamic therapy, ultrasound possesses the benefits of superior tissue penetration and precise targeting, effectively overcoming the limitations in tissue penetration and therapeutic efficacy for deep tumors encountered in photodynamic therapy. Like the principles of photocatalysis, in many piezoelectric sonosensitizers, the inadequate separation of charges hampers the generation of reactive species. Strategies including surface modification for defect introduction, incorporation of dopants, and creation of heterojunctions have been devised to boost the efficiency of reactive species production 97. Creating a heterojunction between different semiconductors forms an interconnected interface that causes band alignment or a rectifying junction once the Fermi levels are balanced at the boundary. This process prolongs the existence of electrons (e -) and holes (h +), thereby inhibiting their swift recombination. Therefore, developing a ZnO heterojunction sonosensitizer represents an effective strategy to improve SDT 98. Li *et al.* 99 rationally designed a platinum-zinc oxide (Pt-ZnO) sonosensitizer to significantly improve the efficacy of SDT through its inherent bandgap structure and dual-enzyme activities (Figure 6A). The Pt-ZnO features a narrow bandgap (2.89 eV) and an optimal amount of oxygen defects, improving the separation efficiency of electrons and holes and generating ROS under US irradiation (Figure 6D). Concurrently, the Pt-ZnO demonstrates dual enzymatic functions, akin to catalase and peroxidase, which efficiently convert endogenous H_2_O_2_ into oxygen O_2_ and lethal ·OH, thereby significantly potentiating SDT and catalytic therapy. Furthermore, the Pt-ZnO exhibits substantial depletion of glutathione, intensifying oxidative stress. In the end, the Pt-ZnO realizes a triple amplification of ROS, with the generation of ^1^O_2_ and ·OH increasing to 859.1% and 614.4%, respectively, leading to an efficacious sono-catalytic treatment and an impressive tumor suppression rate of 98.1% (Figure 6B and C). Nevertheless, in most scenarios, the magnitude of piezoelectric polarization generated by low-frequency ultrasonic pulses is inadequate for triggering redox reactions. Extensive research has enhanced the piezoelectric properties of ZnO nanomaterials such as Au@P-ZnO nanorods, Pt-ZnO nanorods, and ZnO-CdS nanocomposites. However, fine-tuning their structural and compositional parameters remains challenging 100, 101. Xiao *et al.* constructed flying saucer-shaped nano-heterojunctions (Cu_x_O@ZnO) by coating copper oxide (Cu_x_O) on the surface of polyethylene glycolic ZnO nanoparticles, where Cu_x_O not only participates in forming the heterostructure that enhances the piezocatalytic effect but also exhibits a weak Fenton-like reaction, catalyzing the generation of ·OH from the excess H_2_O_2_ produced intracellularly. *In vitro*, experimental results indicate that the Cu_x_O@ZnO heterostructures can effectively inhibit the growth of colorectal tumors by triggering tumor cell apoptosis and ICD, as well as activating the STING signaling pathway 102. Additionally, ion doping within the lattice is regarded as an effective approach to enhance catalyst performance by altering the electronic structure of the catalyst to generate oxygen vacancies (OVs). OVs serve as charge traps to capture excited-state electrons and holes, improve charge separation, and act as electron donors to increase the density of majority carriers. Certain doped transition metals (such as Fe, Mn, Cu, etc.) exhibit enzyme-mimicking catalytic activity activated by TME conditions to produce ROS 103. Tian *et al.* 104 constructed a biodegradable porous manganese-doped zinc oxide (Mn-ZnO) nanocluster. Manganese doping leads to lattice distortion and increased polarization, generating a wealth of oxygen vacancies (OVs), which enhance piezoelectric catalytic performance and enzymatic activity. Mn-ZnO can also promote ROS production and GSH depletion, significantly facilitating the accumulation of lipid peroxides and GPX4, thereby inducing ferroptosis. This study provides more possibilities for the development of new piezoelectric sonosensitive agents.

ZnO NPs have emerged as a promising platform for gas therapy research. ZnO NPs exhibit intrinsic glutathione peroxidase and glycosidase activities, enabling catalytic decomposition of endogenous (S-nitroso glutathione) and exogenous (β-gal-NONOate) nitric oxide (NO) donors to generate high NO concentrations without external donor supplementation 105. The cascade reaction between NO and ROS produces more lethal reactive nitrogen species, enhancing therapeutic efficacy. Based on this, Wang *et al.* 106 designed C-Z@CM nanoparticles by doping Cu-based MOF material (Cu-MOF) with ZnO to obtain Cu-ZnO and coating tumor cell membranes on the surface of Cu-ZnO to obtain C-Z@CM (Figure 6E). The camouflage of cell membranes prevents C-Z@CM from being cleared during the blood circulation process, permitting the C-Z@CM to aggregate at the tumor site through homologous membrane targeting. The presence of ZnO catalyzes endogenous GSNO at the tumor site to continuously produce high concentrations of NO without the need to introduce exogenous NO donors. Copper ions in C-Z@CM catalyze Fenton-like reactions, converting H₂O₂ to ·OH while depleting GSH, achieving potent tumor suppression (Figure 6F and H).

In summary, ZnO nanomaterials have the potential for multiple applications in cancer therapy, including PDT, SDT, and gas therapy. They exhibit selective toxicity towards cancer cells by generating ROS and disrupting mitochondrial membrane potential, leading to caspase activation and apoptosis. However, challenges such as the limited light penetration depth into tumor tissues, finite ROS production, wide ZnO bandgap, and insufficient charge separation efficiency remain. These issues can be potentially resolved through advanced strategies such as surface modification, ion doping, and heterojunction creation.

### Application of ZnS in tumor therapy

Metabolic reprogramming in cancer cells results in inadequate ATP production, impairing the transmission of proliferative signals through downstream signaling pathways, since all kinase-mediated phosphorylation events require ATP as an essential substrate 107. The Warburg effect describes a distinct metabolic phenotype in cancer cells, characterized by preferential utilization of aerobic glycolysis over mitochondrial oxidative phosphorylation (OXPHOS) compared to normal cells 108. Recent research has focused on energy suppression strategies 109; however, the identification of metabolic compensation mechanisms indicates that single-pathway inhibition or partial energy blockade is insufficient to effectively disrupt cancer cell energy metabolism for therapeutic applications 110. Therefore, simultaneous targeting of both glycolysis and OXPHOS to achieve comprehensive energy depletion represents a promising and innovative therapeutic strategy 111. ZnS has emerged as an effective energy metabolism inhibitor in cancer therapy. This section discusses the tumor-inhibitory mechanisms of ZnS and recent advancements in related material development.

**Figure 7:**
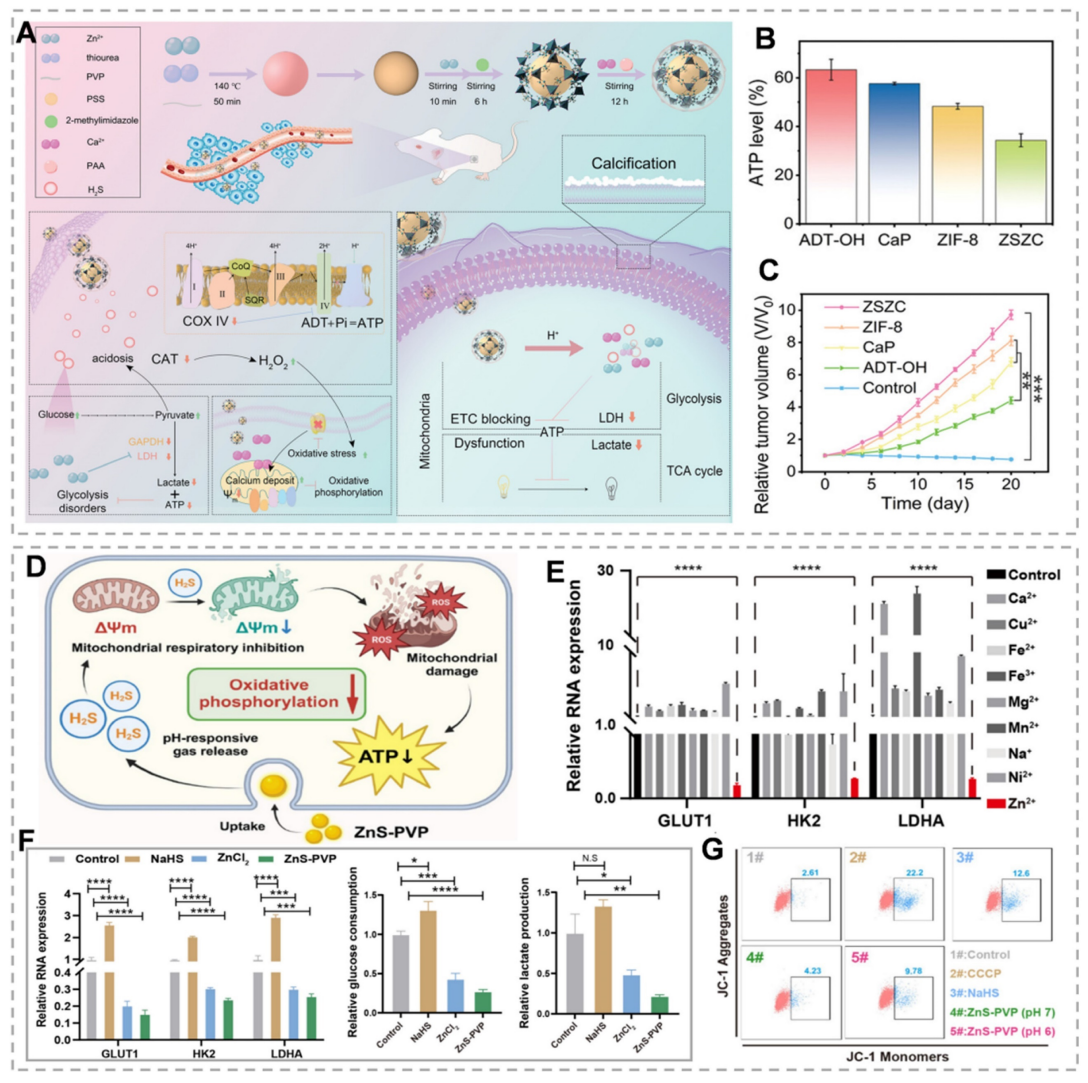
(A) The manufacturing of ZSZC nanoparticles and the schematic illustration of energy barrier treatment. (B) The ATP concentration of 4T1 cells varies with different treatment durations. (C) Relative tumor volume. (D) Diagrammatic representation of mitochondrial damage mediated by H2S and the consequent reduction in energy expenditure. (E) Inhibition of glycolysis by different metal ions. (F) Levels of glycolysis-related gene RNA, glucose, and lactose in Patu8988 cells. (G) Assessment of mitochondrial dysfunction in cells using the JC-1 dye through flow cytometry. **(A-C)** Reproduced with permission from 50, Copyright 2024, Wiley-VCH GmbH. **(D-G)** Reproduced with permission from 117. Copyright 2024, Elsevier B.V.

Gas therapy, recognized as an environmentally friendly cancer treatment modality, has attracted considerable research interest owing to its synergistic enhancement of conventional therapies, including chemotherapy, PDT, immunotherapy, and SDT 112. H₂S, a crucial gaseous signaling molecule, demonstrates dose-dependent dual effects. At low concentrations, H₂S promotes tumor cell proliferation, migration, and other oncogenic processes. However, at higher concentrations, H₂S significantly suppresses cancer progression by triggering apoptosis, arresting the cell cycle, and causing uncontrolled intracellular acidification 113. As an ETC inhibitor, H₂S suppresses cytochrome c expression and induces mitochondrial membrane potential disruption, thereby impairing mitochondrial function, enhancing cellular glucose uptake, and causing irreversible acidification 114. Zn²⁺ effectively downregulates glycolysis-related genes (GLUT1, HK2, and LDHA) via the PI3K-Akt-mTOR-HIF-1α signaling pathway, consequently inhibiting lactate dehydrogenase and glyceraldehyde-3-phosphate dehydrogenase activity 115. Therefore, the Zn²⁺/H₂S combination demonstrates the potential for dual therapeutic effects, particularly in tumor growth inhibition. Recent studies have validated the dual energy-disrupting capabilities of ZnS 116. For example, Liu's team demonstrated that among nine essential bioactive cations, Zn^2+^ exhibits significant glycolysis inhibition (Figure 7E). They synthesized ZnS-PVP nanoparticles and employed immunofluorescence (IF) staining and quantitative real-time PCR to analyze glycolysis-related gene expression (GLUT1, HK2, and LDHA). Results revealed significantly reduced expression of these genes at both protein and mRNA levels in the ZnS-PVP group compared to the ZnCl₂ control (Figure 7F). Treatment of Patu 8988 cells with NaHS and ZnS-PVP for 24 hours increased JC-1 monomer green fluorescence, indicating mitochondrial dysfunction (Figure 7G). Combined with glycolysis inhibition, this establishes a dual-target therapeutic strategy 117. Additionally, H₂S specifically inhibits tumor cell catalase activity, resulting in intracellular ROS accumulation. The buildup of ROS leads to mitochondrial damage and the release of mitochondrial DNA, which in turn activates the cGAS/STING pathway. Intracellular Zn²⁺ further enhances cGAS catalytic activity and amplifies cGAS/STING signaling. H₂S disrupts mitochondrial homeostasis, promoting intracellular Ca²⁺ influx 118. As Ca²⁺ overload induces oxidative stress and mitochondrial dysfunction, rapid Ca²⁺ bursts can alter mitochondrial function, addressing limitations of H₂S gas therapy, including slow onset and instability 119. Yang *et al.* 50. developed ZnZC nanoparticles capable of inducing triple mitochondrial damage, dual glycolysis interference, and multifunctional therapy via acidification and calcification (Figure 7A). ZnS encapsulation within ZIF-8 and CaP minimizes acidic solution exposure, prolonging H₂S release duration. JC-1 probe monitoring revealed that the ZSZC group showed the most severe mitochondrial membrane potential disruption among all treatments. H₂S's catalase inhibition characteristic facilitates calcium overload-induced oxidative stress elevation and maintenance (Figure 7B). *In vivo* studies demonstrated that ZSZC nanoparticle treatment provided the most significant tumor growth inhibition, indicating that simultaneous OXPHOS and glycolysis targeting is the most effective approach (Figure 7C). This energy blockade strategy offers novel insights for developing bioenergetic inhibition-based therapies.

**Figure 8:**
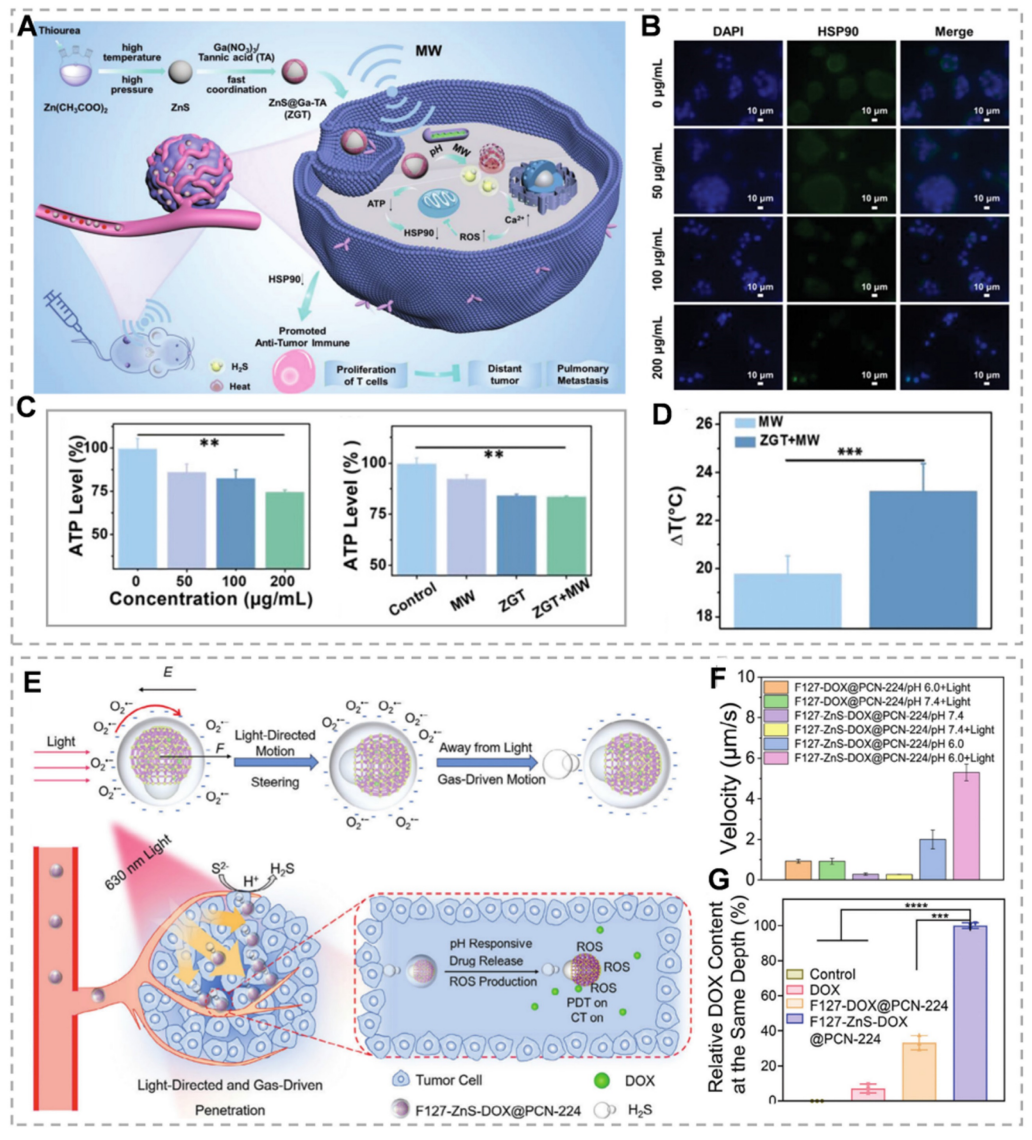
(A) Schematic diagram of the synthesis of ZnS@Ga-TA nano-modulator and its synergistic MWTT anti-tumor effect. (B) Expression of HSP90 in 4T1 cells treated with ZGT nano-modulators at different concentrations. (C) Intracellular ATP concentrations in cells with different treatments. (D) The net temperature rises in real-time infrared thermography of tumors in different groups. (E) The photo-guided and gas-driven movement mechanism of nanorobots in the acidic tumor microenvironment. (F) The average movement speed of different NPs under non-illumination and illumination conditions (n=4). (G) Measure the relative DOX content at the same tumor depth of 105 mm.** (A-D)** Reproduced with permission from 122, copyright 2024, Wiley-VCH GmbH. **(E-G)** Reproduced with permission from 123, copyright 2024, Wiley-VCH GmbH.

ZnS demonstrates potential applications in microwave thermal therapy (MWTT), an emerging clinical modality that targets tumors through thermal effects and antitumor immune response activation. However, complete tumor ablation is often unachievable, and post-ablation heat shock protein 90 (HSP90) upregulation can induce thermotolerance in sublethally damaged tumor regions 120. H₂S-mediated mitochondrial dysfunction inhibits ATP production, consequently downregulating ATP-dependent HSP90 expression. Furthermore, ZnS released under acidic and neutral conditions mediates microwave thermal effects, establishing a foundation for MWTT applications 121. Meng's team developed a ZnS@Ga-tannic acid (ZGT) nanomodulator by capping a gallium metal-phenolic tannate (Ga-TA) network on ZnS nanoparticles. This nanomodulator significantly reduces HSP90 levels and induces caspase-dependent apoptosis (Figure 8A-C). *In vivo* studies demonstrate that ZGT-mediated MWTT combined with H₂S gas significantly inhibits primary tumor growth (Figure 8D). This gas-compensated MWTT strategy shows significant potential for improving tumor ablation therapy outcomes 122. ZnS NPs produce H₂S under acidic conditions. Beyond its intrinsic mechanisms, H₂S gas offers additional therapeutic potential when combined with other modalities. While nanoparticles can respond to various stimuli for targeted delivery, achieving simultaneous rapid movement and precise deep tumor penetration remains challenging. To address these limitations, Wang's team developed the F127-ZnSDOX@PCN-224 nanorobot. They selected the zirconium-based porphyrin MOF PCN-224, which can generate ROS under red light, and used the heterojunction effect of PVP to couple with ZnS to prepare ZnS/PCN-224-based Janus nanoparticles (ZnS-PCN 224) via an interface-induced self-assembly strategy, which were loaded with DOX and then encapsulated by the amphiphilic biocompatible polymer Pluronic F-127. (Figure 8E). Under red light irradiation, negatively charged nanoparticles generate asymmetric O₂^-^ production, creating a local electric field. This field exerts electrostatic forces (F) on negatively charged nanoparticles, propelling them away from the light source. In the acidic TME, ZnS decomposition generates H₂S, which synergizes with electrostatic forces to propel nanoparticles at enhanced velocities (Figure 8F). This propulsion enables nanoparticle migration from tumor surfaces to deeper regions, enhancing tumor accumulation and penetration (Figure 8G). Nanoparticle trajectory analysis reveals enhanced mobility under acidic conditions with negative phototaxis. This study expands ZnS applications through innovative directional transport strategies 123.

ZnS nanoparticles act as both a sulfur reservoir for subsequent reactions and a sacrificial template due to their porous architecture, enabling efficient nanomedicine loading. Zhang *et al.* 124 constructed a Cu-NS@UK@POx nanoplatform, using ZnS as a sulfur source and template to form a single-atom enzyme containing Cu-N₃S₁ active site, whose strong oxidase activity generates reactive oxygen species (ROS), which, combined with the loaded UK5099 and POx, targets pyruvate metabolism, triggers pyroptosis, and reshapes the immune microenvironment to enhance anti-tumor immune effects. Surface-modified ZnS nanoparticles enable precise tumor targeting and therapy. Mesoporous architectures have been shown to amplify ultrasound absorption, significantly enhancing sonocatalytic efficiency. He *et al.* 125 engineered mesoporous ZnS nanoparticles (mZnS) with exceptional sonocatalytic water-splitting capability. The locally generated H₂ and O₂ polarize tumor-associated macrophages from immunosuppressive M2 to antitumor M1 phenotypes, while alleviating hypoxia-induced CD8⁺ T cell suppression, achieving dual antitumor and immune activation effects in deep-seated tumors. ZnS is widely employed as a quantum dot shell material to improve photostability, quantum yield, and photoluminescence efficiency. Liang *et al.* 126 demonstrated that ZnS-modified CuInS/ZnS quantum dots (ZCIS QDs) function as an integrated theranostic platform, providing dual-modal fluorescence and multispectral optoacoustic tomography imaging capabilities. Doping the ZnS lattice with other metals or nonmetals (e.g., Mn, Cu) can give it new fluorescence emission properties or magnetic properties, expanding its potential applications in multimodal imaging (e.g., fluorescence-magnetic resonance imaging) and thus improving diagnostic accuracy and sensitivity.

ZnS shows significant promise in cancer therapy, particularly through energy deprivation strategies. However, it faces challenges such as rapid degradation, a single therapeutic mechanism, biocompatibility issues, and limited synergy with other treatments. For instance, its fast degradation in acidic environments results in unsustainable release of H₂S and Zn²⁺, hindering long-term efficacy. Its single mechanism struggles against tumor heterogeneity and may cause immune responses or harm healthy tissues. Additionally, unclear synergistic mechanisms with other therapies restrict its potential in combination treatments. To address these issues, optimizing material properties, such as fine-tuning crystal structures and surface modifications can enhance stability and targeting. Expanding therapeutic mechanisms by integrating gene therapy and other approaches could enable multi-mechanism synergy. Strengthening combination strategies, systematically studying synergistic effects, and developing tumor microenvironment-responsive nanoparticles could lead to precise treatment with fewer side effects. These advancements offer new directions for ZnS in cancer therapy.

### Application of ZIF-8 in tumor therapy

The swift progress in nanotechnology has led to the emergence of stimulus-responsive nanomaterials as effective choices for developing precise drug delivery systems. Metal-organic frameworks (MOFs), which consist of organic ligands coordinated with metal ions or clusters, have become a rapidly expanding area of research in biomedical applications. ZIF-8 (Zeolitic Imidazolate Framework-8) is a type of MOF material, formed by the coordination of Zn²⁺ ions with 2-methylimidazole ligands 127. Its application in cancer therapy is primarily attributed to its unique physicochemical properties, including high porosity, tunable pore size, excellent biocompatibility, and sensitivity to pH changes. As a carrier, it can deliver chemotherapeutic drugs to tumor tissues and cancerous regions for selective release 128. ZIF-8 has shown its potential applications in various oncological treatment modalities (e.g., SDT, PDT).

**Figure 9:**
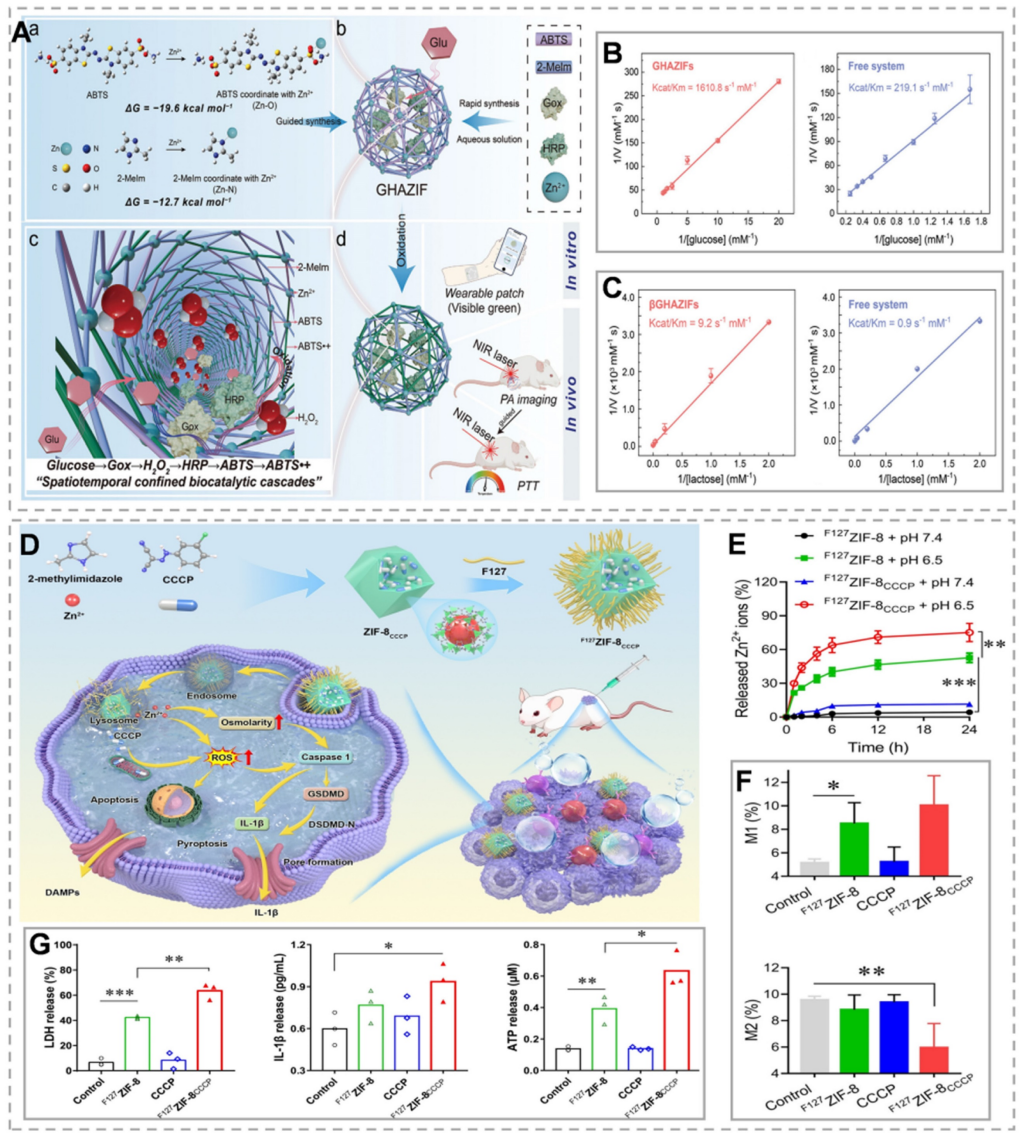
(A) Schematic illustration of the DFT-guided synthesis of GHAZIF and its biological applications. (B) The Lineweaver-Burk plot of GHAZIF, shows the reciprocal of glucose concentration versus the reciprocal of reaction velocity. (C) For GHAZIF, the Lineweaver-Burk plot of the reciprocal of lactose concentration versus the reciprocal of reaction velocity. (D) Schematic illustration of the fabrication of ^F127^ZIF-8_CCCP_ nanoparticles for cancer immunotherapy and the mechanism of pyroptosis induction. (E) Detection of Zn^2+^ release from different materials in response to PH. (F) The relative proportions of M1 and M2 macrophages in mouse splenocytes. (G) The relative levels of LDH, (IL)-1β, and ATP in different groups. **(A-C)** Reproduced with permission from 137, copyright 2024, Wiley-VCH GmbH. **(D-G)** Reproduced with permission from 142, copyright 2023, Wiley-VCH.

The thermal stability of ZIF frameworks is primarily influenced by the chemical composition of the linker molecules used in their synthesis. Additionally, the presence of steric hindrance or electron-withdrawing groups can also affect their stability. ZIFs' porosity is determined by the characteristics of their ligands, solvents, metals, and reaction times. The ligand's structure determines the pores' size and shape; bulky groups might limit pore size, whereas hydrogen bond-forming groups result in smaller pores. Incorporation of polyethylene glycol (PEG) into ZIF-8 nanoparticles during the synthesis of ZIF-8 can significantly improve its dispersion, colloidal stability and loading capacity 129. ZIF-8 demonstrates good stability under physiological pH conditions (∼7.4), but undergoes structural degradation in acidic environments (pH < 6.5). This pH-responsive property enables targeted drug release specifically in the acidic tumor microenvironment 130. For example, Li *et al.* 131 constructed pH-responsive ZIF-8 nanoparticles to deliver the TLR7/8 agonist IMDQ, which was enriched in the liver by its liver-targeting property, and released the drug after uptake by antigen-presenting cells, activating immune cells and inducing antibody production, which synergistically cleared HBV and reduced systemic toxicity. ZIF-8 nanoparticles exhibit low affinity for drugs that lack abundant electronegative groups, such as carboxyl and carbonyl groups, leading to unintended and premature drug release. Additionally, due to the absence of active chemical groups in the structure of ZIF-8, its surface functionalization capability is limited. Using cell membranes to coat the surface of ZIF-8 drug delivery nanosystems is a popular and efficient method. By enabling homologous targeting and achieving sustained drug release, this approach overcomes the limitations of small molecule chemotherapeutic medicines' poor water solubility and non-targeting, resulting in localized chemotherapy effects 132. Xu *et al.* 133 constructed (R837 + OXA)@ZIF-8@CCM (ROZM) nanoparticles. The encapsulation of ZIF-8 achieved effective loading of both drugs, resulting in drug loading rates of 8.09 ± 0.44% and 19.86 ± 0.14% for oxaliplatin and imiquimod, respectively. The modification of the cell membrane changed the surface properties of the nanoparticles, resulting in a negative charge on the surface of ZIF-8 and improved stability, and the cell membrane also endowed the nanoparticles with homologous targeting, and the cellular uptake experiments showed that the RhB-labeled ROZM had a higher intensity of intracellular fluorescence and was better taken up by the tumor cells in the B16-F10 cells compared with the unmodified ROZ. Similarly, Sun *et al.* 134 developed erythrocyte membrane-camouflaged zinc-phenolate nanocapsules (RMP@Cap) incorporating aPD-L1-loaded ZIF-8. The degradation-released zinc ions activate the cGAS-STING pathway, while mitoxantrone-triggered pyroptosis contributes mitochondrial DNA to amplify this signaling. Concurrently, the erythrocyte membrane coating enhances tumor accumulation, and aPD-L1 relieves T-cell suppression. These coordinated actions create a STING-mediated cascade that potently amplifies anti-tumor immunity. Research has revealed that biomolecules, encompassing amino acids, nucleotides, and peptides, can engage in competitive interactions with metal-organic ligands, thereby offering the potential to adjust the catalytic performance of MOF@enzyme hybrid systems 135. A characteristic shared among these biomolecules is the presence of functional groups such as amines, phosphates, or carboxylic acids at either the N- or C-termini. These groups facilitate targeted interactions with the MOF's constituent blocks, enabling the expedited assembly of MOFs in aqueous conditions 136. For instance, Zhang's team prepared GHAZIFs nanoparticles by encapsulating enzymes within MOFs regulated by sulfonic acid-functionalized signal substrates (Figure 9A). The sulfonic acid groups of ABTS adsorb Zn^2+^ ions through charge interactions, forming Zn-O coordination bonds. This rapidly modulates the synthesis of MOFs around Gox and HRP, forming dual-enzyme/substrate and triple-enzyme/substrate MOFs. The biocatalytic efficiency of these MOFs is 7.4 and 10.2 times higher than that of the free system, respectively (Figure 9B and C) 137. ZIF-8 nanoparticles are extensively utilized as drug carriers, capable of loading molecules, enzymes, DNA, and proteins while enabling pH-responsive controlled release. However, their role as carriers is often emphasized, overshadowing the intrinsic therapeutic and immunotherapeutic effects of ZIF-8 itself 138. ZIF-8 nanoparticles (NPs) inherently trigger apoptosis alongside necrosis and ICD, effectively initiating *in situ* immunization through the rapid release of inflammatory molecules and DAMPs. ICD is a form of programmed cell death where dying tumor cells release DAMPs, such as calreticulin and ATP. These DAMPs play a vital role in antigen presentation and can further stimulate a coordinated immune response 139. ZIF-8 NPs release pH-sensitive Zn²⁺, leading to a sudden increase in ion concentration and intracellular osmotic pressure, which activates the caspase-1/gasdermin D-dependent pyroptosis pathway. Beyond recruiting immune cells to activate antitumor immunity, ZIF-8 NPs also reprogram the immunosuppressive TME by shifting macrophages from the M2 to M1 phenotype, showcasing their intrinsic immunostimulatory properties (Figure 9F). The process can be enhanced by incorporating modulators into the nanoparticles 140, 141. For instance, Lin *et al.* 142 loaded the mitochondrial depolarizer CCCP into ZIF-8 nanoparticles (Figure 9D) and found that it further intensified the pyroptosis process, accompanied by ICD induction, pyroptosis activation, and the release of DAMPs (Figure 9G). Compared to ZIF-8 nanoparticles, ZIF-8CCCP nanoparticles exhibited a more pronounced pH-responsive controlled release process, with a Zn ion release rate of up to 75% within 24 hours under acidic conditions (Figure 9E). This result might be explained by the swelling effect induced by the incorporation of CCCP.

ZIF-8 can serve as a photoresponsive nanomaterial that, upon exposure to UV light, produces O₂⁻, facilitating photocatalytic-driven PDT 143. Similar to ZnO, ZIF-8's wide bandgap (Eg = 5.1 eV) restricts its photocatalytic activity to the UV range (λ <400 nm), limiting therapeutic applications 144. To solve this problem, researchers have developed various doping strategies to adjust the bandgap and extend the light response range. For example, Fan *et al.* 145 introduced Ag-doped ZIF-8 with a bandgap of 3.06 eV, demonstrating vigorous photocatalytic activity under visible light (< 490 nm). Similarly, Mai *et al.* 146 synthesized Fe-doped ZIF-8 with a bandgap of 2.2 eV, broadening the spectral response range of ZIF-8 to the visible light region (< 563 nm). However, these doping approaches only shift the excitation threshold of ZIF-8 photosensitizers to the shorter-wavelength visible light range (< 563 nm), leaving longer-wavelength visible light unabsorbed and unused by ZIF-8. Furthermore, while NIR light penetrates biological tissues more deeply than UV-visible light, its low photon energy prevents direct excitation of ZIF-8 photosensitizers. As a result, there is a pressing need for light wave conversion technologies to transform long-wavelength NIR light into shorter-wavelength UV-visible light for ZIF-8 excitation 147. Yang *et al.* 148 developed lanthanide-doped nanoparticles (LDNPs) coated with Fe/Mn-doped ZIF-8 (LDNPs@Fe/Mn-ZIF-8) for second NIR-II imaging-guided synergistic PDT/CDT. The incorporation of Fe²⁺/Mn²⁺ significantly narrows the bandgap of the ZIF-8 photosensitizer from 5.1 eV to 1.7 eV, extending its excitation threshold into the visible light range (λ = 650 nm). Fe/Mn-ZIF-8 can degrade in the tumor microenvironment, releasing Fe²⁺/Mn²⁺ ions that generate ·OH through Fenton-like reactions, enabling CDT. At the same time, the degradation of Fe/Mn-ZIF-8 enhances the nanosystem's tumor-specific NIR-II imaging capabilities, offering precise guidance for CDT/PDT. The doping strategy of graphene quantum dots (GQDs) can also be applied to ZIF-8. MOF nanoparticles can be readily modified with various materials. The epoxy and carboxyl groups on GQDs enable the functionalization of MOFs, resulting in MOF/GQD composite nanoparticles that exhibit photothermal properties. Zhu *et al.* 149 developed DOX-ZIF-8/GQD nanoparticles, which not only facilitate intracellular drug release in the acidic environment of cancer cells but also enable photothermal therapy through the photothermal effect of GQDs under near-infrared radiation. As a novel photosensitizer, reduced graphene oxide (rGO) is also commonly used for MOF doping, which not only has a higher photothermal effect compared with GQDs but also provides a better template for the *in situ* growth of ZIF-8 nanoparticles. As a larger carrier, it enhances the intracellular concentration of ZIF-8 NPs, enabling more effective destruction of cancer cells 150. Ma *et al.* 151 designed a multifunctional nanoplatform of BSArGO@ZIF-8 NSs, in which ZIF-8 NPs were uniformly grown on the rGO surface to form small-sized and well-dispersed complexes, which were subsequently modified by reduced ascorbic acid and bovine serum albumin. Under near-infrared (NIR) light irradiation, rGO absorbs light energy and converts it into heat energy, which increases the local temperature. The experimental data showed that the photothermal conversion efficiency of BSArGO@ZIF-8 NSs was about 22.5% under 808 nm NIR irradiation, and at the same time, the combination with ZIF-8-mediated ionic interference therapy (IIT) could produce a synergistic effect and enhance the therapeutic effect.

ZIF-8 materials also exhibit sonodynamic properties. The unsaturated zinc-nitrogen (Zn-N) active sites on the surface of ZIF-8 NCs facilitate ligand-enhanced electron transfer to the metal charge transfer band, transitioning from the highest occupied molecular orbital (HOMO) to the lowest unoccupied molecular orbital (LUMO). Under the stimulation of US irradiation, these sites can activate adsorbed O_2_ and H_2_O molecules on the surface, converting them into ROS damaging to tumor cells 152. *In vivo* experiments have shown that ZIF-8 NCs, both bioactive anticancer agents and sonosensitizers, demonstrate high tumor suppression efficiency (84.6%) 153. Hollow nanomaterials can enhance the cavitation effect, thereby significantly amplifying the SDT effect. Recently, Liu *et al.* 154 designed MOF-derived double-layer hollow nanoparticles (DHMS). These nanoparticles were created by fully etching ZIF-8 in ZIF-8@mSiO₂ to form DHMS, with Mn ions grown on both the inner and outer surfaces of the mSiO₂ layer. The hollow porous structure of DHMS enhances the cavitation effect, which amplifies the SDT effect, while Mn²⁺ is oxidized by holes under ultrasonic irradiation, which significantly improves the electron-hole separation and promotes the generation of ROS. Chen *et al.* 155 investigated the construction of cell-derived nanorobot SonoCu, which achieved the synergistic effect of SDT and cuproptosis by encapsulating copper-doped ZIF-8 in macrophage membranes, effectively inhibiting tumor growth. ZIF-8 has been found to induce cell death by modulating the P13K pathway, thereby promoting autophagy. Conversely, the autophagy process can also facilitate the degradation of ZIF-8, leading to the production of Zn^2+^ and ROS 156. Additionally, ZIF-8 is non-toxic to the human body and features pores that facilitate the attachment of probes to disease sites. Consequently, ZIF-8-based composite materials have been widely utilized in multiple imaging techniques, such as magnetic resonance imaging (MRI), photoacoustic imaging (PAI), fluorescence imaging (FI), and X-ray computed tomography (CT). These materials improve cancer detection and treatment by facilitating targeted drug delivery to cancer cells and enabling more accurate tumor imaging.

In summary, ZIF-8 is a multifunctional nanomaterial with significant potential in tumor therapy. However, it faces several challenges, including difficulties in controlling pore size, structural instability during functionalization, poor *in vivo* stability, unverified biocompatibility, imprecise drug release regulation, and insufficient imaging targeting. Addressing these limitations through precise synthesis and surface functionalization techniques could enable better control over pore size, structure, and functionalization, thereby enhancing material performance. Additionally, incorporating protective layers or polymers to improve *in vivo* stability, conducting thorough biocompatibility studies, optimizing drug-loading efficiency, and implementing targeted modifications could further advance its applications in drug delivery and cancer therapy. With continued research, ZIF-8 holds promise for expansion into diverse fields such as biosensing, brain disease treatment, and tissue engineering, paving the way for multifunctional therapeutic and diagnostic applications.

### Application of other Zn²⁺ materials in tumor treatment

**Figure 10:**
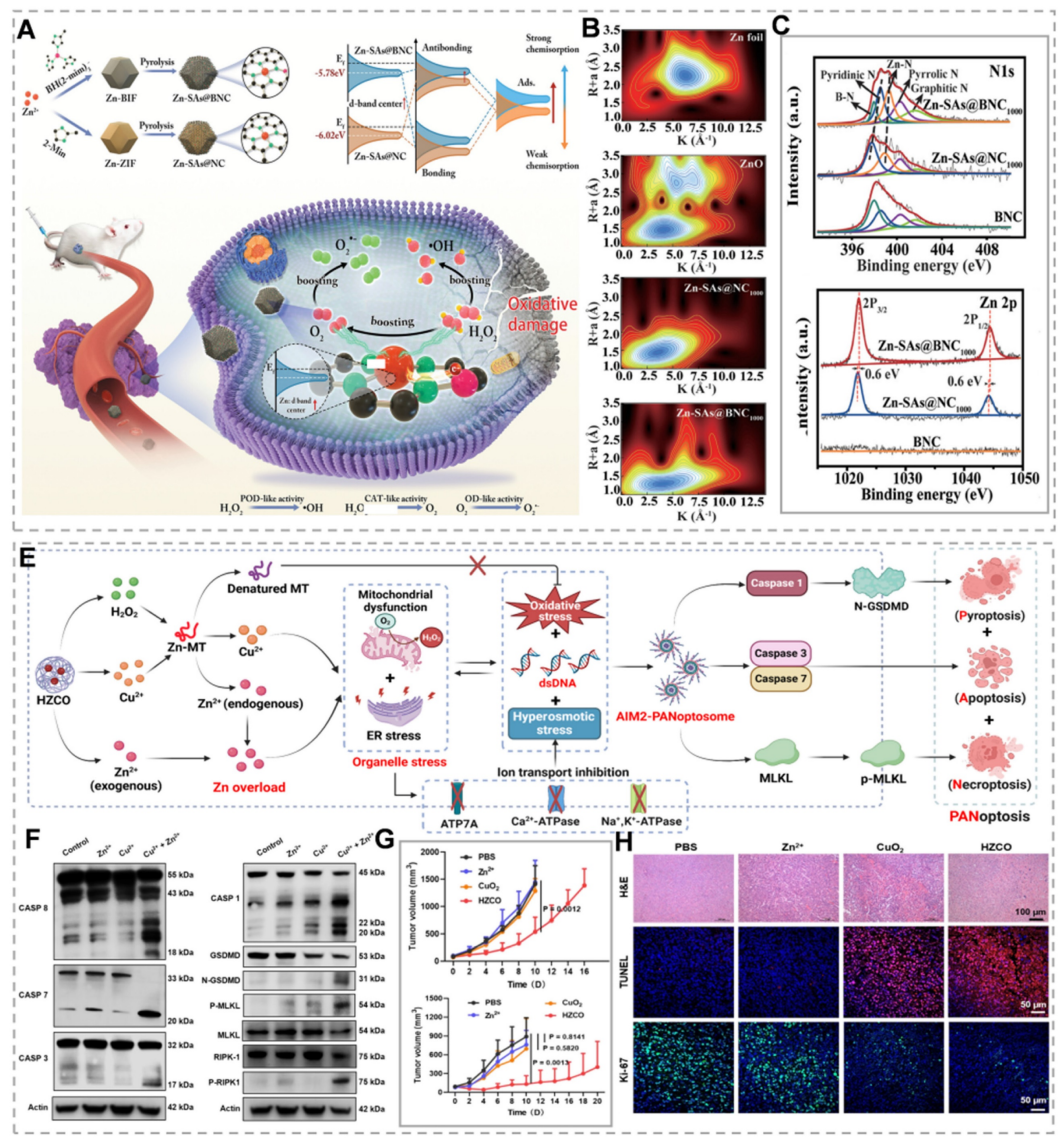
(A) Construction of Zinc Single-Atom Nano-Enzymes and Schematic of Their Tumor Therapeutic Mechanisms. (B) Wavelet transformation of Zn K-edge EXAFS of Zn foil, ZnO, Zn-SAs@NC1000, and Zn-SAs@BNC1000. (C) B doping has a regulatory effect on the electron density of Zn atoms and the d-band center. (E) Schematic diagram of HZCO-induced PANoptosis. (F) The impact of different treatments on the expression of PANoptosis-II-related proteins in 4T1 cells. (G) Tumor volume size transformation after different treatments. (H)Validation of the anti-tumor effects of HZCO. **(A-C)** Reproduced with permission from 159, copyright 2020, Wiley-VCH GmbH. **(E-H)** Reproduced with permission from 171, copyright 2024, American Association for the Advancement of Science.

Beyond the previously discussed conventional Zn-based materials, Zn²⁺ demonstrates therapeutic potential through alternative modalities. Zn-based single-atom nanozymes (SAzymes) demonstrate exceptional biocompatibility and have been extensively investigated for their potential diagnostic and therapeutic applications 157. The fully occupied 3d¹⁰ electron configuration of Zn²⁺ imparts enhanced stability and biocompatibility. However, this electronic configuration limits Zn²⁺ electron mobility and catalytic activity in Fenton-like reactions. Typically, the catalytic center's coordination environment can be optimized through modification of the primary or secondary coordination spheres in metal single-atom sites by incorporating heteroatoms or creating carbon vacancies 158. Furthermore, synergistic interactions among multiple catalytic centers significantly contribute to enhanced catalytic performance. Zhang *et al.* 159 prepared boron-doped Zn-based SAzymes (Zn-SAs@BNCx, x=800, 900, 1000, and 1100 °C) by carbonizing zeolite-like Zn-based imidazolium-boron skeletons at different temperatures. (Figure 10A). The formation of B-N bonds generates a localized electric field, modulates the d-band center position, and facilitates Zn→N→B electron transfer, consequently enhancing the Zn²⁺ oxidation state (Figure 10B). These modifications enhance H₂O₂ and O₂ adsorption and activation by Zn-SAs@BNC1000, thereby boosting its multi-enzyme catalytic activity. Boron doping enhances the catalytic efficiency (Kcat/Km) of Zn-SAs@BNC1000, endowing it with 24.81-, 32.37-, and 13.98-fold higher peroxidase-, oxidase-, and catalase-like activities, respectively, compared to the undoped counterpart, compared to the undoped material. Furthermore, Zn-SAs@BNC1000 demonstrated significant tumor-killing ability both *in vivo* and *ex vivo*. (Figure 10C). Li *et al.* 160 designed an atom pair engineering of Zn-SA/CNCl-SAzyme by simultaneously constructing a Zn-N4 site as a catalytic site and a Zn-N4Cl1 site as a catalytic modulator. The Zn-N₄Cl₁ catalytic modulator enhances H₂O₂ adsorption and facilitates Zn-N₄ catalytic center re-exposure, consequently accelerating reaction kinetics. This SAzyme also demonstrates significant peroxidase-like activity and effectively suppresses tumor growth in both *in vitro* and *in vivo* settings.

Zn²⁺, owing to its cationic nature, can form chelation complexes with diverse therapeutic agents. This chelation not only modulates the spatial conformation of drug molecules but also improves their stability and bioavailability, consequently enabling targeted therapeutic outcomes. Wang *et al.* 161 fabricated nanostructures with distinct morphologies via coordination-driven self-assembly of metal ions (Zn²⁺, Fe²⁺, Mg²⁺) with l-phenylalanine. They demonstrated that Ph-Zn nanosheets exhibited superior cellular uptake efficiency, with significantly enhanced internalization in dendritic cells (DCs) following short-term starvation (STS) treatment compared to other groups. Ph-Zn nanosheets effectively remodeled the immunosuppressive TME, increasing tumor-infiltrating DCs and CD8⁺ T cell populations while elevating intratumoral cytokine levels (TNF, IFNγ, and IL-6), ultimately suppressing tumor growth and inducing apoptosis. Zn²⁺ can also undergo *in situ* chelation with therapeutic agents within the tumor microenvironment. Zhang's team designed Zn^2+^-doped disulfide (DSF)-loaded mesoporous silica nanoparticles (DSF@Zn-DMSN). Zn^2+^ and DSF were released simultaneously in the mildly acidic tumor microenvironment, forming toxic Zn^2+^ chelates via an *in situ* chelation reaction. These complexes induce significant apoptosis, generate DAMPs, and activate autophagy-mediated DAMP release, thereby enhancing ICD 162. Qiu *et al.* 163 developed zinc-liganded lipid nanoparticles (A-CaO₂-Zn-LNP), which co-delivered CaO₂ and the STING agonist diABZI-2 with a DPA-containing lipid-liganded skeleton and Zn²⁺ was released to synergize with calcium overload in inducing immunogenic cell death while chelating diABZI-2 activates the cGAS-STING pathway, and combines with CaO₂ to alleviate the acidity and hypoxia of the tumor microenvironment, remodels the immunosuppressive microenvironment, and enhances anti-tumor immunity and long-term memory. Yang *et al.* 164 designed zinc cyclic di-AMP nanoparticles (ZnCDA), which encapsulate the STING agonist cyclic di-adenosine monophosphate (CDA) through zinc ion nanocoordination polymers to prolong the CDA cycle time and target tumors efficiently. ZnCDA can destroy tumor vascular endothelial cells and enhance its accumulation in tumor cells, and can also target tumor-associated macrophages to regulate their antigen processing and presentation capabilities. Chelation-based strategies provide innovative therapeutic approaches for disease management. In addition, Zn²⁺ has applications in gene therapy, where Zn²⁺ stabilizes the catalytic core structure of the DNAzyme through coordination, binds to the phosphoric acid backbone or base to neutralize the negative charge, and facilitates the folding of the DNAzyme into an active conformation, thereby enhancing the efficiency of specific cleavage of the target RNA 165, 166. Wu *et al.* 167 used Zn^2+^ to activate a DNA enzyme capable of cleaving glucose transporter protein 1 (GLUT1) mRNA to induce tumor-specific energy depletion through tumor starvation therapy. Yao *et al.* 168 constructed a Mn/Zn bimetallic MOF (PMZH nanoplatform) that releases Zn²⁺ under acidic conditions in the tumor microenvironment to activate the CRISPR plasmid and reverse T-cell depletion through modulation of the cGAS-STING pathway, as well as to induce ROS generation to synergistically kill tumor cells.

Zinc ions demonstrate therapeutic potential not only as monotherapies but also through synergistic combinations with other metal ions, yielding outcomes that surpass their individual effects. PANoptosis represents a recently identified programmed cell death mechanism that integrates features of multiple cell death pathways, including pyroptosis, apoptosis, and necroptosis. This process incorporates key elements from multiple cell death pathways, and involves diverse molecular activation events and a complex regulatory signaling network orchestrated by the PANoptosome complex 169. This mechanism can initiate a robust inflammatory response and release DAMPs, consequently activating systemic anti-tumor immunity 170. Building on this concept, Cheng *et al.* 171 developed hydrated hyaluronic acid (HHA)-modified Zn-CuO_2_ nanoparticles (HZCO) (Figure 10E). The bioactive HZCO nanoparticles facilitate targeted delivery of Cu²⁺ and Zn²⁺. Cu²⁺ displaces Zn²⁺ from MT, elevating intracellular free Zn²⁺ levels. This leads to Zn²⁺ overload and subsequent mitochondrial dysfunction. *In vitro* studies demonstrate that this combination simultaneously cleaves pyroptosis-related proteins (caspase-1 and GSDMD) and apoptosis-related proteins (caspase-3/7/8) while inducing phosphorylation of necroptosis-related proteins (RIPK1 and MLKL). Formation of ASC specks is observed, with co-localization of RIPK1, caspase-8, and caspase-1, confirming PANoptosome assembly (Figure 10F). *In vivo* studies reveal enhanced therapeutic efficacy of the Zn²⁺/Cu²⁺ combination in tumor treatment (Figure 10G). In the 4T1 breast cancer mouse model, Zn²⁺/Cu²-based HZCO nanoparticles demonstrate superior tumor growth inhibition compared to Zn²⁺ or CuO₂ alone, and are evidenced by reduced tumor cell proliferation (decreased Ki-67 expression) and increased apoptosis (enhanced TUNEL fluorescence) (Figure 10H). Similar broad-spectrum antitumor effects are observed in CT26 and B16F10 subcutaneous tumor models. These findings demonstrate that coordinated apoptosis-pyroptosis integration effectively eliminates tumor cells while eliciting robust immune responses. Building on similar principles, Cheng *et al.* 172 developed zinc-nickel hydroxide (ZnNi(OH)₄) nanosheets. Ni²⁺ and Zn²⁺ induce paraptosis and pyroptosis respectively, and their combination establishes a paraptosis-pyroptosis positive feedback loop through synergistic interactions. Ni²⁺ amplifies Zn²-induced pyroptosis through dual mechanisms: (1) paraptosis induction reduces cellular resistance to Zn²-mediated pyroptosis, and (2) increased intracellular free Zn²⁺ concentrations trigger pyroptotic cell death via Zn²⁺ overload. This dual mechanism effectively eliminates tumor cells and stimulates robust inflammatory responses, enhancing antitumor immunity and immunotherapy efficacy.

## Conclusion and Prospects

Zn²⁺ plays key roles in cellular metabolism, signaling and oxidative stress regulation. Their metabolic regulatory functions are closely related to the survival and drug resistance mechanisms of tumor cells. In this paper, we comprehensively review the progress of the mechanism of action and application of Zn²⁺ and their nanomaterials in tumor therapy. In recent years, Zn-based nanomaterials (e.g., ZnO_2_, ZnO, ZIF-8, ZnS, etc.) have shown considerable potential in cancer therapy due to their unique physicochemical properties and TME responsiveness. These materials not only target the release of Zn²⁺ to interfere with tumor energy metabolism, but also significantly enhance therapeutic efficacy by inducing oxidative stress, activating the immune response, and synergizing with other therapies (photodynamic, acoustic, and immunotherapeutic). Despite significant progress in laboratory studies, the clinical translation of these materials still faces complex challenges.

Firstly, balancing biocompatibility and toxicity is the core issue for the clinical translation of Zn-based nanomaterials. The toxicity of zinc-based nanomaterials (e.g., ZnO, ZIF-8) is closely related to their morphology, size and environmental transformation. It has been shown that ZnO nanomaterials with different structures (e.g., nanoparticles, short/long nanorods) exhibit differentiated biological effects, and their toxicity mechanism mainly involves size-dependent cellular uptake, ROS generation, and sulfation and other environmental transformation processes. Their biosafety can be effectively enhanced by surface modification (e.g., NAC retardation), composite construction and precise size regulation. Studies on the safe dosage of zinc-based nanomaterials are still relatively limited, and results from different studies vary. Zinc-based nanomaterials have a promising application in tumor therapy, and although there is a lack of successful clinical trial cases, a large number of preclinical studies have demonstrated that zinc-based nanomaterials have great potential for cancer diagnosis, targeted drug delivery and therapy.

Second, the pronounced heterogeneity of the tumor microenvironment presents significant challenges for the targeted delivery and tissue penetration of Zn-based nanomaterials. Tumor regions frequently demonstrate characteristic features including acidic pH, hypoxic conditions, and elevated interstitial pressure, which can compromise nanoparticle stability and induce premature drug release or functional inactivation. For instance, the dense ECM can impede nanomaterial penetration into deeper tumor regions, thereby limiting therapeutic efficacy to superficial areas. Furthermore, the antioxidant defense systems of tumor cells, such as overexpression of glutathione due to metabolic reprogramming (e.g., the Warburg effect), may attenuate the oxidative stress effects induced by Zn²⁺. Designing intelligent nanocarriers that can actively adapt to microenvironmental changes, such as enzyme-responsive or mechanically driven penetration strategies, is key to improving therapeutic efficacy.

Third, the long-term stability and controlled degradability of Zn-based materials require substantial optimization. Numerous Zn-based nanomaterials (e.g., ZIF-8, ZnO₂) undergo structural disintegration under physiological conditions through biomolecule adsorption or ion exchange processes, resulting in premature Zn²⁺ release. This phenomenon not only diminishes therapeutic efficacy but may also promote drug resistance development. For instance, although ZIF-8's rapid degradation in acidic microenvironments facilitates targeted drug release, its degradation byproducts may disrupt the tumor immune microenvironment by promoting infiltration of immunosuppressive cells, such as M2 macrophages. Furthermore, inadequate nanomaterial stability in systemic circulation can cause nonspecific biodistribution and elevated off-target effects. The development of composite coatings (e.g., biomimetic cell membrane modifications) or cross-linked architectures could extend material circulation half-life while enabling spatiotemporal responsiveness.

Finally, significant technical challenges remain in achieving effective integration of mechanisms for multimodal synergistic therapy. While Zn-based nanomaterials are frequently integrated with photothermal therapy, sonodynamic therapy, or immunotherapy to enhance therapeutic outcomes, the spatiotemporal synergistic mechanisms among these modalities remain incompletely understood. For instance, photothermal therapy may aggravate tumor hypoxia, thereby compromising the effectiveness of oxygen-dependent sonodynamic therapy. Moreover, Zn²-induced immune activation may be counterbalanced by immune cell toxicity when combined with chemotherapeutic agents. Furthermore, the absence of standardized dosing protocols and temporal coordination among therapeutic modalities may result in inconsistent efficacy and cumulative adverse effects. Future research should focus on leveraging computational modeling and real-time monitoring technologies to develop dynamic treatment regulation systems, enabling genuine precision synergy.

Building on these challenges, future development of Zn-based nanomaterials should focus on advanced material modification and delivery strategies. pH-responsive polymer coatings such as poly (β-amino ester) could enable tumor-specific Zn²⁺ release while minimizing systemic exposure. Cell membrane camouflage techniques utilizing platelet or cancer cell membranes may enhance tumor targeting through natural homing mechanisms while extending circulation half-life. For improved tissue penetration, enzyme-responsive surface modifications combined with size-reducing strategies could facilitate deeper tumor penetration. The integration of real-time monitoring components like MRI contrast agents would allow for treatment guidance and dose optimization. Furthermore, the development of dual-responsive systems combining pH and redox sensitivity could achieve spatiotemporal control of Zn²⁺ release, while zwitterionic coatings may help balance material stability with microenvironmental responsiveness. Zinc ions and their nanomaterials show revolutionary therapeutic prospects in the field of tumor metabolism intervention and redox regulation through unique multi-targeting mechanisms of action, providing an important direction for the development of a new generation of precise anti-cancer strategies.

## Figures and Tables

**Table 1:** Comparison table of signaling pathways and molecular mechanisms of zinc ion-induced cell death

Type of mechanism	Main pathways of action	Key signaling pathways	Signal conduction details	Core molecule / structure
Necrotic	Ion homeostasis imbalance, mitochondrial dysfunction, and endoplasmic reticulum stress.	MT-MTF-1 axis, ROS oxidative stress pathway, ER stress pathway.	Zn²⁺ displaces MT binding sites, free zinc ↑ Mitochondrial ROS ↑ → membrane damage, dsDNA release PERK pathway activation → cell rupture	Zn²⁺, MT, MTF-1, mitochondria, ROS, dsDNA, ER.
Apoptosis	Mitochondrial pathway	Mitochondrial electron transport chain damage, caspase cascade reaction.	Zinc ions accumulate in mitochondria via ZnT2, inhibiting the electron transport chain → ROS burst → mitochondrial membrane damage, releasing Cyt-c → activation of caspase-9/3.	Mitochondria, ROS (O²-/H₂O₂/·OH), Cyt-c, Apaf-1, caspase-9, caspase-3.
	Death receptor pathway	TNF-α-mediated exogenous apoptotic pathway.	Zinc ions downregulate caspase-8 and activate TNFR1 → caspase-8 to cut caspase-3 to initiate apoptosis.	caspase-8, caspase-3, TNFR1.
Pyroptosis	Classical pathway (caspase-1/GSDMD)	NF-κB pathway, NLRP3 inflammatory vesicle signaling pathway.	Zinc ion-induced ROS activation, NF-κB → NLRP3 inflammatory vesicle assembly → caspase-1 cleaves GSDMD to form membrane pores.	NLRP3, ASC, caspase-1, GSDMD, NF-κB.
	Alternative pathway (caspase-3/GSDME)	Mitochondrial apoptotic pathway and pyroptosis switching pathway.	Zinc ions activate caspase-3 via mitochondrial pathway → cleave GSDME to generate membrane pores (GSDME-positive cells only).	GSDME, caspase-3, Bcl-2 family proteins.
	mtDNA-AIM2 inflammatory vesicle pathway	cGAS-STING pathway, AIM2 inflammatory vesicle signaling pathway.	Zinc ion induces ROS damage to mitochondria to release mtDNA → mtDNA activates cGAS-STING and binds AIM2 → caspase-1 cuts GSDMD.	mtDNA, AIM2, cGAS, STING, caspase-1.
	NOX1-ROS pathway cross-talk	NADPH oxidase signaling pathway.	Zinc ions upregulate NOX1 → catalyze NADPH production of O²- → enhance ROS → synergistically activate NF-κB and inflammatory vesicles.	NOX1, NADPH, ROS.

**Table 2:** Summary of unique mechanisms and combined therapeutic strategies for zinc-based nanomaterials

Zinc-based nanomaterials	Common mechanisms	Unique Mechanisms	Combination therapy strategies and applications
ZnO2	Zn²⁺ release disrupts tumor energy metabolism. Induces oxidative stress. Interferes with zinc homeostasis and ROS cyclic self-amplification to induce apoptosis/fibroblastification. Activates immune responses, and modulates pathways such as cGAS/STING to enhance anti-tumor immunity.	Acidic degradation produces Zn²⁺ and H₂O₂ synergistic Fenton reaction. Inhibition of ZnT1 enhances zinc overload. Concentration-dependent induction of pyroptosis/apoptosis.	PDT, SDT, immunotherapy (αPD-1), combined photothermal-chemical.
ZnO		Semiconductor photocatalysis for ROS production. Piezoelectric effect for ROS enhancement. Noble metal modification for broadening the light response. Mimicking enzyme activity.	Combined photoacoustic therapy (PPDT/SDT), gas therapy (catalyzed NO generation), and photothermal therapy (PDA coating).
ZnS		Release of H₂S inhibits the mitochondrial respiratory chain and synergizes with zinc ions to cause energy deprivation. Activation of cGAS/STING and Ca²⁺ overload in acidic environments.	Energy-immunotherapy, microwave thermotherapy, SDT, light-responsive nanorobotics.
ZIF-8		pH-responsive release of Zn²⁺-induced focal death/ICD. Porous drug-carrying controlled release. Surface Zn-N participation in acoustic dynamics. Metal doping to expand the photoresponse.	SDT, photodynamic-chemical combination, immunotherapy (activation of DC/T cells), photothermal-ion combination.
Other Zn²⁺ materials		Metal ion synergy (e.g., Zn²⁺ + Cu²⁺/Ni²⁺). Ligand self-assembly regulates the immune microenvironment	Catalytic therapy (e.g., Zn-SAs@BNC1000), immune-combination therapy (remodeling TME), and multimodal cell death induction.

**Table 3:** Different application areas and mechanisms of action of ZnO-NPs

Application Area	Mechanism of Action	Therapeutic Effect	Research Progress	Ref
Chemotherapy sensitizer	Inhibition of P-gp expression improved cell membrane permeability	Increased intracellular drug accumulation, enhanced chemotherapy efficiency	Further research is needed to determine effective concentrations and safety	173
Photosensitizer	Induction of ROS generation under UV irradiation	Attacking cell membranes, facilitating the entry of antitumor drugs into cells	Further research is needed to verify the efficacy	174
Drug carrier	Targeted delivery of anticancer drugs	Improved targeting and therapeutic efficacy of drugs	Development of various metal oxide nanoparticles	175
Biosensor	Used as a biosensor	Provides new strategies for treating hematological tumors	Good antitumor effects were observed *in vitro* cell experiments	176
Cell cycle arrest	Preventing cells from entering mitosis	Inhibition of tumor cell proliferation	More large-scale randomized controlled trials are needed	177
Induction of oxidative stress	Generation of excessive ROS	Leading to the apoptosis of tumor cells	Studying the cytotoxicity of ZnO NPs	178
Regulation of protein and gene expression	Activation of Caspase, influence on MT1 gene expression	Exerting tumor suppressor activity	Studying the synthesis methods of ZnO NPs	179
Regulation of Zn²⁺ homeostasis	Dissolution producing Zn²⁺, affecting intracellular homeostasis	Leading to mitochondrial dysfunction and apoptosis	Studying the biocompatibility of ZnO NPs	180
Promotion of mitophagy	Activation of PINK1/Parkin-mediated mitophagy	Inducing cell autophagy	Studying the clinical application of ZnO NPs	83
Regulation of mitochondrial membrane potential	Decreasing MMP, leading to a decline in ATP levels	Mitochondrial dysfunction	Studying the cytotoxicity of ZnO NPs	181

## Abbreviations

ATP: Adenosine triphosphate
ROS: Reactive oxygen speciesl
H₂O₂: hydrogen peroxide
GSH: glutathione
PTT: Photothermal therapy
SDT: Sonodynamic therapy
LDH: Lactate dehydrogenase
α-KGDHC: α-ketoglutarate dehydrogenase
ETC: Electron transport chain
O₂⁻: superoxide anions
MT: metallothionein
EMT: Epithelial-mesenchymal transition
MMP: Mitochondrial membrane potential
OH: hydroxyl radical
Cyc-c: Cytochrome c
TNF-α: Tumor necrosis factor-α
GSDMD: Gasdermin D
ICD: Immunogenic cell death
CDT: Chemodynamic therapy
SiRNA: Small interfering RNA
GPX4: Glutathione peroxidase 4
EMC: Extracellular matrix
WTp53: wild-type p53
HSPs: Heat shock proteins
MDT: Molecular dynamic therapy
¹O₂: singlet oxygen
HO2: superoxide radicals
NIR: Near-infrared
PPDT: Piezo-photodynamic therapy
OXPHOS: Oxidative phosphorylation
MWTT: Microwave thermal therapy
MOFs: Metal-organic frameworks
ZIF-8: Zeolitic Imidazolate Framework-8
